# Efficient detection and typing of phage-plasmids

**DOI:** 10.1128/mbio.03000-25

**Published:** 2026-02-09

**Authors:** Karina Ilchenko, Remy A. Bonnin, Eduardo P. C. Rocha, Eugen Pfeifer

**Affiliations:** 1 Université Paris-Saclay, INRAE, AgroParisTech, MICALIS, Jouy-en-Josas, France; 2 Center for Immunology of Viral, Auto-immune, Hematological and Bacterial diseases, Université Paris-Saclay, Inserm, CEA, (IMVA-HB/IDMIT/UMRS1184), Fontenay-aux-Roses 568414 , Le Kremlin-Bicêtre, France; 3 Bacteriology-Hygiene Unit, Bicêtre Hospital Assistance Publique-Hôpitaux de Paris, Le Kremlin-Bicêtre, France; 4 Associated French National Reference Center for Antibiotic Resistance: Carbapenemase-Producing Enterobacteriaceae., Le Kremlin-Bicêtre, France; 5 Institut Pasteur, Université Paris Cité, CNRS, UMR3525, Microbial Evolutionary Genomics 555089, Paris, France; 6 Université Paris-Saclay, INRAE, MetaGenoPolis 569277 , Jouy-en-Josas, France; Montana State University, Bozeman, Montana, USA

**Keywords:** phage-plasmids, phages, plasmids, genomics, mobile genetic elements, hidden Markov models

## Abstract

Phage-plasmids (P-Ps) are temperate phages that replicate as plasmids during lysogeny. Despite their high diversity, they carry genes similar to phages and plasmids. This leads to gene exchanges and to the formation of hybrid or defective elements, which limits accurate detection of P-Ps. To address this challenge, we developed tyPPing, an easy-to-use method that efficiently detects and types P-Ps with high accuracy. It searches for distinct frequencies and sets of conserved proteins to separate P-Ps from plasmids and phages. tyPPing’s strength comes from both its precise predictions and its ability to systematically type P-Ps, including the assignment of confidence levels. We tested tyPPing on several databases and a collection of incomplete (draft) genomes. While predictions rely on the quality of assemblies, we detected high-quality P-Ps and experimentally proved them to be functional. Compared to other classification methods, tyPPing is designed to detect distinct P-P types and surpasses other tools in terms of sensitivity and scalability. P-Ps are highly diverse, making the systematic identification of new types a difficult task. By combining tyPPing with other tools, however, we show a valuable foundation for addressing this challenge. How to use tyPPing and other approaches is documented in our GitHub repository: github.com/EpfeiferNutri/Phage-plasmids/.

**IMPORTANCE:**

Mobile genetic elements, such as phages and plasmids, are diverse and drive bacterial evolution through horizontal gene transfer. Phage-plasmids, of which many carry antibiotic resistance genes or virulence factors, are both phages and plasmids and have life cycles of temperate phages and plasmids. This makes accurate classification difficult as current computational tools typically classify them as one or the other. We addressed this problem by developing tyPPing, a new and highly precise method, to systematically identify, separate, and catalog phage-plasmids. We demonstrated that tyPPing is highly accurate and broadly compatible. It provides a reliable foundation for all future studies involving phages and plasmids, ranging from agriculture environments to pathogenic strains of clinical settings.

## INTRODUCTION

Phage-plasmids (or prophage-plasmids, short P-Ps) challenge the traditional dichotomy between a bacteriophage (or phage), which is infectious and often lytic, and a plasmid, which is a stable, autonomously replicating genetic element. P-Ps are defined as temperate dsDNA phages that typically lack integrases and replicate episomally in bacterial hosts as plasmids. Contrary to integrative temperate phages, the episomal state allows P-Ps to exist as multiple copy elements (per cell). This polyploidy has been reported to promote heterozygosity (1). As plasmids, P-Ps rely on partitioning systems to ensure their proper segregation to daughter cells (during cell division), and they may encode addiction modules (toxin/antitoxin systems [2, 3]), facilitating genetic stability. Signals such as DNA damage or quorum sensing molecules have been described (4–6) to cause the switch to the lytic cycle, ultimately initiating the production of new progeny and lysis of the host. Most of our current understanding of P-Ps stems from studies on two well-characterized phages, P1 (7) and N15 (8). However, in recent years, a growing body of research has begun to shed new light on their biology and broader impact (9–12).

Separating P-Ps from other phages and plasmids is challenging given their dual nature. For clarity, we will refer to them as P-Ps and differentiate them from other phages and other plasmids (which will be just called phages and plasmids). P-Ps are reported to be diverse and widespread across bacteria (9). They encode a variety of intriguing traits, such as virulence factors (9, 13) and antibiotic resistance genes (ARGs) (14), with the latter being rare in phages (14, 15). Yet, clinical strains often harbor ARGs that are encoded on P-Ps, suggesting that they have a substantial role in their dissemination (14, 16, 17). P-Ps have homologs in plasmids, which facilitates recombination and potentially the acquisition of ARGs. They can spread ARGs via lysogenic conversion and remain infective as phages (once arrived in a new host) (14). We therefore postulate that P-P detection is crucial to understand ARG transmission. Furthermore, as P-Ps also possess homologs in phages, they drive a gene flow between phages, plasmids, and P-Ps (18), positioning them as a key exchange point between mobile genetic elements (MGEs). P-Ps exhibit evolutionary plasticity and may evolve to plasmids and to integrative prophages (18). These dynamics suggest a continuous nature of P-Ps, phages, and plasmids and underscore substantial challenges in the precise classification of P-Ps.

No specific tool has been developed to effectively detect P-Ps, with most current methods focusing on the detection of integrative prophages, plasmids, and other MGEs (19–24). We noted that P-Ps are often mis-annotated, solely as phages or plasmids. To tackle this, we screened plasmid and phage databases for P-Ps and created a first P-P catalog to address their diversity (9). In our approach, we annotated plasmid sequences with phage profiles and used random forest models to class them, and screened phages for plasmid functions. We complemented our search with an exhaustive literature review and added cases that were missed by the two approaches. By using the gene repertoire relatedness of P-Ps, we grouped them into well-related types: AB, P1, N15, and five SSU5-related groups. Most of these (91.7%) are predicted to infect Enterobacterales species. The other P-Ps were either singletons (unique P-Ps that are not related to others) or clustered into sparsely related communities with members that are frequently detected (approx. 1 out of 2) in monoderm species (*Actinomycetia*, *Bacilli*, and *Clostridia*) (9). We found that the P-P types have unique characteristics related to their gene repertoires (core and accessory), genomic sizes, and evolutionary histories. Yet, our first approach, despite detecting many P-Ps, faces important drawbacks. First, the detection requires time-consuming and labor-intensive (semi-automated) screens that are prone to overlook very diverse P-Ps due to legacy models and profiles. Second, this method is poorly scalable and poorly applicable on draft genomes or metagenomic sequences, limiting the assessment of P-P diversity. Lastly, because it was designed to spot P-Ps broadly without considering the distinct type-specific characteristics, it frequently causes inconsistencies when new members are added to already defined types (e.g., during database updates).

We aimed to improve the detection of P-Ps and their subsequent typing. For this, we developed and present here “tyPPing,” a user-friendly method tailored for the distinct P-P types. tyPPing is capable of simultaneously detecting and typing the most prevalent P-P types. It searches for conserved P-P proteins and uses their frequency and distinct repertoires (compositional sets) to accurately separate P-Ps from other MGEs. Moreover, tyPPing assigns confidence scores (high to low), which facilitates the detection of *bona fide* P-Ps and atypical, debatable cases. We trained tyPPing on our most recent P-P data set (18) and compared its performance to that of our first approach (9) using complete genomes of phages and plasmids, and draft genomes of bacteria. Finally, we compared tyPPing to other classification tools, such as vConTACT v2 (25) and geNomad (19), and we discuss the performance of all approaches, highlighting the benefits and downsides in the strategies of detecting P-Ps.

## MATERIALS AND METHODS

### Collecting sequences of phages, plasmids, and P-Ps

We collected two genomic databases on different time points, and the sequences were retrieved from the non-redundant NCBI RefSeq database (26). The first data set was generated in March 2021. It contains 21,550 sequences assigned as plasmids and the complete genomes of 3,725 phages. We will refer to this data as “03/21.” We detected in this data set 1,416 P-Ps (18) (see Table S1). The second data set, retrieved in May 2023 (“05/23”), contains 38,051 sequences, all labeled as plasmids. In comparison to 03/21, 17,540 sequences are new in 05/23 (Table S1).

### Similarity of MGEs assessed by gene repertoire relatedness

To compare MGE pairwise, we computed their weighted gene repertoire relatedness (wGRR), as done previously (9, 18, 27). Specifically, to compute the wGRR, we count the number of best bi-directional hits (BBH) between two MGEs, weight them by protein sequence identities, and normalize the score to the protein number encoded by the smallest genome. To obtain the number of BBHs, we compute pairwise protein alignments using MMseqs2 (v.14.7e284) (28) and keep all hits with an E-value under 10^−4^ with >35% sequence identity covering at least >50% for both protein sequences. The wGRR was then calculated with the following formula:


$$wGRR(A/B)=\frac{\sum_{i}^{P}id\left(A_{i};B_{i}\right)}{min\left(A;B\right)}$$

Here, A_i_ and B_i_ represent the i-th BBH pair out of a total of P pairs, with the gene count of the smaller element being min (A, B), and the sequence identity between the BBH pair is id (A_i_, B_i_). A wGRR = 0 means no matching genes, and wGRR = 1 means all protein sequences of the smaller element are identical to the BBH in the other element.

### Detection and grouping of phage-plasmids by multi-model gene repertoire clustering (MM-GRC)

To detect P-Ps in plasmids of 05/23, we used our initial approach to detect P-Ps, which employs the multiple-models approach and combines it with a gene-repertoire clustering (MM-GRC) (9). Briefly, we annotated genes with phage-like functions in plasmids using 2,583 phage-specific hidden Markov models (HMMs) with HMMER v3 (29). A hit was considered positive if at least 50% of the profile was covered with a domain i-E-value lower than 10^−3^ (as used in MacSyFinder (30)). The profiles are grouped into six functional categories: (i) structure; (ii) lysis; (iii) packaging, maturation/assembly, and DNA injection; (iv) recombination, regulation, and DNA metabolism; (v) unknown; and (vi) others. Counts per functional category were used to compute annotation profiles, which served as feature tables for 10 distinctly trained random forest models. These models were used to predict scores ranging from 0 (plasmid-like) to 1 (phage-like). Scores of the 10 models (one score per each model) were averaged, and elements with values >0.5 were kept. All sequences with sizes >10 kb (smallest reported dsDNA phage) and <300 kb were considered putative P-Ps. We aimed to minimize false positives by excluding very large sequences (>300 kb) that likely represent mega plasmids (31) co-integrated with an integrative prophage. This step resulted in 887 P-Ps in 05/23 (Table S1). The here-used phage profiles, the random forest models, and a customized R script are made available in our GitHub repository (github.com/EpfeiferNutri/Phage-plasmids/).

To type P-Ps, we used the wGRR, which we computed between P-Ps from 03/21 and 05/23. Specifically, P-Ps from 05/23 were assigned to types if they had a wGRR of ≥0.5 to a P-P from 03/21 and at least 50% of the genes were homologs. In case of multiple matches, the type of the best hit (highest wGRR) was assigned. Since cp32 P-Ps are not detected by the random forest models as P-Ps (9), we added 39 cp32 P-Ps manually to the 05/23 P-P list using the abovementioned criteria. In total, we detected 926 P-Ps in 05/23 that were not already present in 03/21 (Table S1). Lastly, we placed the 926 P-Ps into well-related (*n* = 498), sparsely related groups (*n* = 150), and P-P singletons (*n* = 278).

### Curation of the cp32 P-Ps

Circular sequences of sizes of approx. 32 kb were described as P-Ps in *Borrelia burgdorferi* (32). However, MM-GRC did not detect them as such since the protein profiles failed to annotate the phage functions. Thus, in previous work, we placed cp32 sequences (*N* = 87) in a non-curated P-P group since we could not detect functions typical for phages (9). Nonetheless, in a recent study, virion structure proteins (capsid, fibers, tail tube, and needles) were described in genomes of five cp32s (NC_012158, NC_012156, NC_012154, NC_012152, and NC_012149) (33). We used these cp32 sequences to define a well-related cp32 P-P group. Briefly, we compared the five cp32 P-Ps with 422 plasmids from *Borrelia* and *Borreliella* (of 03/21) using the wGRR. We kept sequences having at least 50% of genes as BBHs with one of the five cp32s and a wGRR of 0.6 or higher, resulting in 83 matches (Fig. S1). We then considered only elements between 27 kb and 37 kb to exclude too short and too long sequences as they are probably impaired in the lytic phage cycle. In total, 70 elements were kept and added into a cp32 group in 03/21 (Table S1).

### P-P profiles derived from conserved protein families

We generated 763 signature profiles specific for 10 P-P groups (Table S2). For this, we extracted conserved protein sequences from P-Ps of the well-defined groups (of 03/21): AB_1, P1 subgroup 1 (P1_1), P1 subgroup 2 (P1_2), N15, SSU5_pHM2, pMT1, pCAV, pSLy3, pKpn, and cp32. Then, in multiple steps, we generated multiple sequence alignments, used these to produce HMM profiles, and then characterized them.

#### Extraction of conserved protein families

We used PPanGGOLiN (v1.2.63) (34) (default) on sequences of the 10 P-P groups to characterize their pangenomes. PPanGGOLiN utilizes MMseqs2 (28) to cluster protein sequences into protein families with at least 80% identity and a sequence coverage of 80%. Then, it calculates a presence/absence matrix of the protein families in genomes and categorizes them according to their frequencies into persistent (found in most P-Ps), shell (present in an intermediate number of P-Ps), and cloud (found in only a few P-Ps). We selected the highly frequent protein families (persistent or quasi-core) to generate specific P-P profiles.

#### Generation and curation of global multiple sequence alignments

We used MAFFT (v7.487) (35) to generate global multiple sequence alignments of each protein family that we manually inspected and removed unaligned overhanging regions (solely at the N- and/or C-termini parts).

#### Conversion to HMM profiles and functional annotation

Then, we used the curated alignments to generate HMM profiles with HMMER (v3.3.2) (29) and computed a sequence score for each profile, as it is typically done for protein families in PFAMs (36). This score is defined as the minimum score that includes all sequences used in the alignment. We then annotated the P-P profiles using Prokaryotic Virus Remote Homologous Groups (PHROGs) (37) and the database of geNomad (19) by comparing one representative protein sequence to these profiles. Functional categories were assigned for positive hits having E-value ≤ 10⁻⁴ and cover at least 50% of the profile (Table S2; Fig.S4).

#### Diversity and specificity of HMM profiles

To assess profile sequence diversity, we calculated the number of effective sequences, defined as NEFF values (38). Briefly, we used HH-suite3 (39) (v3.3.0) to compute these values with the “hhmake” function. NEFF values were extracted from the headers of the resulting “.*hhm”* files. NEFF scores measure the average diversity at each position within a sequence alignment. To compute them, the average genetic randomness (Shannon entropy) is computed for small sections of the alignment. For each section, a specific NEFF value is computed that is the exponential of the Shannon entropy. The final NEFF score is the average of all individual NEFF values per alignment (Fig. S2).

To assess the specificity of the HMMs, we counted the numbers of P-Ps, phages, and plasmids matching them and computed two specificity scores: (i) P-P score and (ii) P-P-type score (Table S2; Fig. S3). A positive hit was assigned if a profile matches a protein sequence and passes the sequence score. If a protein sequence matched multiple HMMs, we counted only the profile with the best hit (lowest E-value) per protein sequence. The P-P score was computed as follows:


$$(2)\;PP\text{-}score=\frac{\#PP}{\#PP+\#Ph,Pl}$$

#PP is the number of P-Ps detected (by this profile), and #Ph,Pl represents the total number of phages and plasmids hit (by the same profile). Scores close to 1 suggest high specificity for P-Ps, whereas values closer to 0 indicate a low specificity.

To compute the P-P-type score, we applied the following equation:


$$(3)\;PP\text{-}type\text{-}score=\frac{\#PP\left(type\right)}{\#PP\left(type\right)+\#Ph,Pl,PP\left(Rest\right)}$$

#PP(type) is the number of P-P genomes of a specific type that were detected by the profile (passed the sequence threshold). #Ph,Pl,PP(Rest) is the number of profile matches against phages, plasmids, and other P-P types. A score close to 1 indicates a high specificity in terms of identifying the correct P-P type, and a score close to 0 indicates the opposite.

### Detection of P-Ps and confidence scoring of P-Ps with tyPPing

We used the P-P type-specific HMM profiles to develop tyPPing. Details on how to use it, along with its full documentation, are available in our GitHub repository (https://github.com/EpfeiferNutri/Phage-plasmids). We used sequences of phages, plasmids, and P-Ps of 03/21 to define specificity thresholds (Fig. S5 to S9) and sequences of 05/23 to validate its performance. To use tyPPing, a profile-to-protein comparison table (using all 763 profiles) needs to be computed for protein sequences encoded by a given genome sequence with HMMER (v3.3.2) (29). In addition, information on the genome/contig sizes and the protein and genome IDs needs to be provided in two tables. Then, by using the provided R script, tyPPing predicts if the given sequence is a P-P and assesses the confidence level of the prediction. In its workflow, tyPPing first computes two parameters for a given sequence: (i) the number of conserved P-P proteins and (ii) which conserved P-P proteins matched this sequence. Next, it compares these parameters to the thresholds of MinProteins and Composition.

Briefly, for MinProteins, tyPPing searches a given sequence for highly conserved P-P proteins and keeps only profile matches that pass the sequence score threshold. If the number of matches is higher than the type-specific cutoff, the given sequence is kept. Cutoffs were set to detect complete P-Ps and filter out plasmids, phages, or truncated variants (Fig. S5 and S6).

For Composition, tyPPing considers profile matches as positive if protein sequences cover the matched profiles at least by 50% (Fig. S7). Next, tyPPing compares if the detected set of protein sequences matches P-P-specific sets (or compositions). To account for genetic diversity, we tested how this approach detects P-Ps when protein sets are not identical (if ≤25% of proteins per composition or a specific number of proteins is allowed to be missing). We selected those cutoffs that allow the detection of P-Ps and exclude other MGEs (phages, plasmids, and P-Ps of other types) (Fig. S8). By this, tyPPing retains only sequences having a similar organization as found in genomes of reported P-Ps. The protein sets, their sizes, and allowed number of missing proteins are listed in Table 1 and are retrievable from our GitHub repository.

**TABLE 1:** Characteristics of the 10 well-defined P-P types^*a*^

P-P type	Key example	Virion morphology (of key example)	Taxa of the host (most frequent)	Conserved protein families (count)	MinProtein (cutoff)	Composition sizes (cutoff for missed proteins)	Size range (kb)
AB_1	SSU5 (distant)	*Siphovirus*	*Acinetobacter baumannii*	94	55	62–94 (7)	101–122
cp32	φBB-1	*Myovirus* (*elongated head*)	*Borrelia burgdorferi*	32	18	24–32 (6)	28–33
N15	N15	*Siphovirus*	*Enterobacteriaceae* (*Klebsiella pneumoniae*)	43	7 (24)	16–43 (9)	40–66
P1_1	P1	*Myovirus*	*Enterobacteriaceae* (*Escherichia coli*)	71	49	43–71 (11)	72–125
P1_2	D6	*Myovirus*	*Enterobacteriaceae* (*E. coli*)	84	46	71–84 (17)	79–104
pCAV	SSU5	*Siphovirus*	*Enterobacteriaceae* (*K. pneumoniae*)	73	30	42–73 (3)	103–118
pKpn	SSU5	*Siphovirus*	*Enterobacteriaceae* (*K. pneumoniae*)	89	38	75–89 (2)	99–125
pMT1	SSU5	*Siphovirus*	*Enterobacteriaceae* (*Yersinia pestis*)	92	50	47–92 (6)	85–115
pSLy3	SSU5	*Siphovirus*	*Enterobacteriaceae* (*E. coli*)	95	47	60–95 (3)	85–134
SSU5_ pHCM2	SSU5	*Siphovirus*	*Enterobacteriaceae* (*Salmonella enterica*)	90	71	81–90 (3)	94–126

^*a*^ This includes reported examples, along with their virion morphologies, taxa of (frequent) host if P-Ps were found in host of different species, number of conserved protein families (computed by PPanGGOLiN [34]), thresholds for tyPPing, and size ranges.

Lastly, tyPPing assigns confidence levels for predictions (High, Medium, and Low). For this, it tests whether a given sequence matches MinProteins, Composition, and a type-specific size range. We determined the size ranges by taking the mean size ± 3× standard deviations of sequences of curated P-P types (excluding P-Ps with atypical sizes, Fig. S9). The criteria for the confidence levels are listed in Table 2. Briefly, a high confidence is assigned when a given sequence matches MinProteins, Composition, and fits the size range of the predicted P-P type. A medium confidence indicates that this element is identified at least by MinProteins or Composition and exceeds the size range up to 10% (of the mean size). A low confidence score is assigned to sequences that substantially deviate from the P-P type specific size range (>10%) and are at least detected by MinProteins and/or Composition.

**TABLE 2:** Criteria for confidence levels assigned by tyPPing

Confidence	MinProteins	Composition	Within size range	Remark
High	Yes	Yes	Yes	High confidence P-P, meets all criteria
Medium	Yes	Yes/no	Yes or ± 10%	Meets MinProteins **and/or** Composition, and fits slightly extended size range
	Yes/no	Yes		
Low	Yes	Yes/no	No	As for medium, **but** is substantially smaller or larger
	Yes/no	Yes		
Not detected as P-P	No	No	Not tested	Does not meet criteria

### P-P detection and typing with geNomad and vConTACT v2

We applied geNomad (v1.7.4, database version 1.5) (19) to all 05/23 sequences labeled as “plasmids” (default parameters). From the virus summary table, we considered 1714 sequences as putative P-Ps since they were identified as “phage” (or “virus”) (Table S1). However, it is important to note that geNomad does not perform sequence clustering, meaning it cannot assign types to these putative P-Ps.

To test vConTACT v2 (25), we first clustered all sequences from 05/23 (default settings) with 1,416 P-Ps from 03/21 (18). A sequence was considered a P-P if it grouped with a P-P and was assigned the same type. We tested two clustering cutoffs. A “stricter” approach, where only cases were considered that clustered clearly with P-Ps (referred to as “Clustered” by vConTACT v2). In the second approach, we considered cases that were grouped into overlapping clusters or were categorized as Clustered/Singleton (Clustered, Overlap, and Clustered/Singleton are described in the wiki of vConTACT v2). Upon closer inspection, we noted that vConTACT v2 frequently clustered many plasmids with P-Ps, even though these plasmids did not encode phage genes, that is, they are not true P-Ps. The clustering was based solely on the presence of plasmid-typical genes.

We therefore combined geNomad predictions with vConTACT v2’s clustering analysis. Briefly, we used vConTACT v2 to cluster the 1,714 geNomad-predicted putative P-Ps with the 1,416 P-Ps from 03/21 (after the exclusion of duplicated entries). P-P types were assigned if a sequence grouped with a previously typed P-P (9). A P-P was considered a singleton if vConTACT v2 could not group it with others.

### Detection of P-Ps in the incomplete genome of carbapenem-resistant strains

To screen draft genomes for P-Ps, we modified tyPPing and made its counting of conserved P-P proteins cumulative, that is, we allowed matches even if they span multiple contigs. Additionally, we excluded counts from contigs larger than 300 kb (as these are likely parts of the bacterial chromosome or megaplasmids), those encoding fewer than 3 protein sequences, and those with less than 10% conserved P-P proteins (relative to all proteins encoded on the contig). We first tested this version on 32,798 bacterial assemblies after removing all matches to P-Ps that were identified by tyPPing (with any confidence). We then counted matches to chromosomal regions and plasmids collectively (Fig. S17). We noted that 12–14 distinct N15 profiles had many matches (*N* = 460) to structural gene cassettes that are often detected in *Klebsiella* chromosomes (40). To avoid detecting them as putative P-Ps, we increased the N15 MinProteins cutoff from 7 to 24, for elements that did not pass the Composition.

We then tested the modified version of tyPPing and compared the results to those of MM-GRC (9, 14). For this, we screened draft genomes of the French National Reference center, including P-Ps from our previous work (14), and of three additional strains: a P1_2 in 170D8, a P1_1 in 174J8, and an N15 in 204G7 (Table 3, detailed in Table S4).

**TABLE 3:** tyPPing’s predictions for P-Ps detected in short- and long-read assemblies and complete genomes (resulted from hybrid assembly)

Source	Strain	P-P type	Confidence short-read assemblies	Across “x” contigs	Confidence long-read assemblies	Across “x” contigs	Confidence complete	Packaged in the virion
Pfeifer et al., (14)	163A9	P1_1	High	5	Not detected	0	High	Yes
	166F4	pSLy3	High	2	High	1	Not detected	No
	169C2	N15	High	2	High	2	High	Yes
	169C2	SSU5_ pHCM2	High	1	Not detected	0	Medium	Yes
	170E2	N15	High	2	High	1	High	Yes
	170E2	SSU5_ pHCM2	High	1	Not detected	0	High	Yes
	171A5	N15	High	2	High	2	High	Yes
	171A5	SSU5_ pHCM2	High	1	Not detected	0	High	Yes
	211G7	SSU5_ pHCM2	Medium	3	Not detected	0	Medium	Yes
This work	170D8	P1_2	Not detected	0	Medium	1	High	Yes
	174J8	P1_1	High	1	Not detected	0	High	Yes
	204G7	N15	High	2	High	2	High	Yes

We extracted whole genomic DNA of the bacterial strains and subjected it to sequencing by long reads. Briefly, cells were cultivated in 4 mL LB-Miller medium at 37°C for ~16 h (overnight) with shaking at 250 rpm. After a centrifugation step, DNA from cell pellets was isolated with a modified version of the guanidium thiocyanate method (prior to DNA precipitation, the samples were treated with RNase A at 37°C for 30 min) (41). DNA library preparation (SMRTBell Library 10 kb insert size) and sequencing were done at the Biomics sequencing platform of the Institut Pasteur (C2RT) (Paris, France) with the technology of Pacific Biosciences (PacBio). The obtained reads were assembled by Flye (v.2.7.1-b1590) (42) with default parameters.

### Mitomycin C induction experiments and purification of phage particles

We tested if P-Ps, which we predicted in the strains 170D8, 174J8, and 204G7, are inducible by mitomycin C (MMC). We first cultivated them in 4 mL LB-Miller medium at 37°C for ~16 h (overnight). Stationary cultures were diluted 1:100 and cultivated in new 4 mL LB-Miller medium at 37°C medium for 1 h. MMC (Sigma-Aldrich, St. Louis, United States) was added in final concentrations of 1 μg/mL. At 4 h after MMC addition, samples of 2 mL were taken for PEG precipitation and pelleted. The supernatant was filtered using a 0.22 μm filter and was referred to as phage lysates.

### Extraction of DNA from phage particles and sequencing

To purify phage particles, we treated phage lysates with polyethylene glycol (PEG). For this, we added PEG-8000 in a final concentration of 10% (wt/vol) and NaCl of 0.5 M. The mix was inverted several times and chilled on ice overnight. Next, virions were pelleted at 60 min and 5,000 × *g*, at 4°C, and the supernatant was carefully discarded. Pellets were resolved in 100 uL buffer solution (10 mM Tris HCl, pH 7, 10 mM MgCl_2_). To remove bacterial host DNA and RNA, we treated the samples with 2 U Turbo DNase for 60 min at 37°C and 5 U of RNAse H. DNase and RNase were subsequently inactivated by adding Proteinase K in a final concentration of 0.1 μg/mL and 0.5% SDS at 62°C for 20 min. Viral DNA was extracted using a phenol-chloroform method (43). Briefly, an equal volume of phenol:chloroform:isoamyl alcohol (25:24:1, vol/vol/vol) was added to the sample, mixed by inversion, and centrifuged at 10,000 × *g* for 30 min. The upper aqueous phase was then transferred to a new tube and extracted with an equal volume of chloroform:isoamyl alcohol (24:1, vol/vol) using the same procedure. DNA was precipitated by adding 0.1 volumes of 3 M sodium acetate (pH 5.2) and 2.5 volumes of ice-cold absolute ethanol, followed by overnight incubation at −20°C. The precipitated DNA was collected by centrifugation at 12,000 × *g* for 60 min at 4°C, washed with 500 μL of 70% ethanol (centrifuged at 12,000 × *g* for 15 min at 4°C), and finally resuspended in Tris-HCl buffer (pH 8.0). The DNA concentration was determined using a Qubit fluorometer. For P-Ps from 170D8 and 174J8, library preparation, sequencing (paired end, 150 bp), and quality checks were performed by Eurofins Genomics (Ebersberg, Germany). For 204G7, these steps were prepared on the Biomics sequencing platform of the Institut Pasteur (C2RT) (Paris, France) by short reads (paired end, 250 bp length). Read sets were made available in the European Nucleotide Archive (see the data availability section).

### Hybrid assembly, read processing, and mapping

We removed adapters and low-quality reads with fastp (v0.23.4) (default parameters) (44). We then used them together with long reads (from the PacBio sequencing) and assembled the P-P genomes with Unicycler (v0.5.0) (45). We analyzed the sequences with geNomad (v1.7.4) with default parameters (19) and tyPPing. We then aligned the reads to the assemblies with bwa-mem2 (v2.2.1) (46) and filtered out low-quality alignments with msamtools (v1.1.3) (https://github.com/arumugamlab/msamtools) using the parameters “-l 80 -p 95 -z 80 –besthit.” Mapped reads were counted using the msamtools’ profile function with “--multi=proportional.” To compute an accurate signal from the host sequences, we first mapped the reads on phage and P-P sequences and collected all unmapped reads (= non-phage) with samtools v1.9 (47). For 174J8 and 204G7, we then aligned these non-phage reads on the long-read assemblies to calculate the average read coverage. In total, 98.4% for 174J8 and 99.4% for 204G7 of the non-phage reads mapped on the long-read assemblies. For 170D8, we achieved a higher read coverage (94.9% of the reads) when we mapped the non-phage reads on the hybrid assemblies, instead of the long-read assemblies, as these were of too low quality.

## RESULTS

### Protein profiles specific for P-Ps cover phage and plasmid functions

We aimed to develop a method that is specific and fast in detecting P-Ps. For this, we aimed to use HMMs built from protein sequences. We focused on sequences of prevalent, curated P-P types (according to our classification system [9]) and selected types that have at least 10 members and experimentally validated cases (proven to be both phages and plasmids). We excluded small and sparsely related clusters due to the limited number of available sequences or low homology to experimentally proven P-Ps. Our selection included P-Ps of AB_1, P1_1, P1_2, N15, SSU5_pHCM2, pMT1, pCAV, pSLy3, pKpn, and cp32 (key characteristics of these types are listed in Table 1). Notably, in our previous study (9), cp32 P-Ps, reported as φBB-1 phages making 11.2% of the P-Ps, were not assigned as curated since their lytic genes were unknown. Here, we include them (see Methods, Fig. S1) as in a recent study, the function of genes involved in the virion formation has now been confirmed and annotated (33).

**Fig 1:**
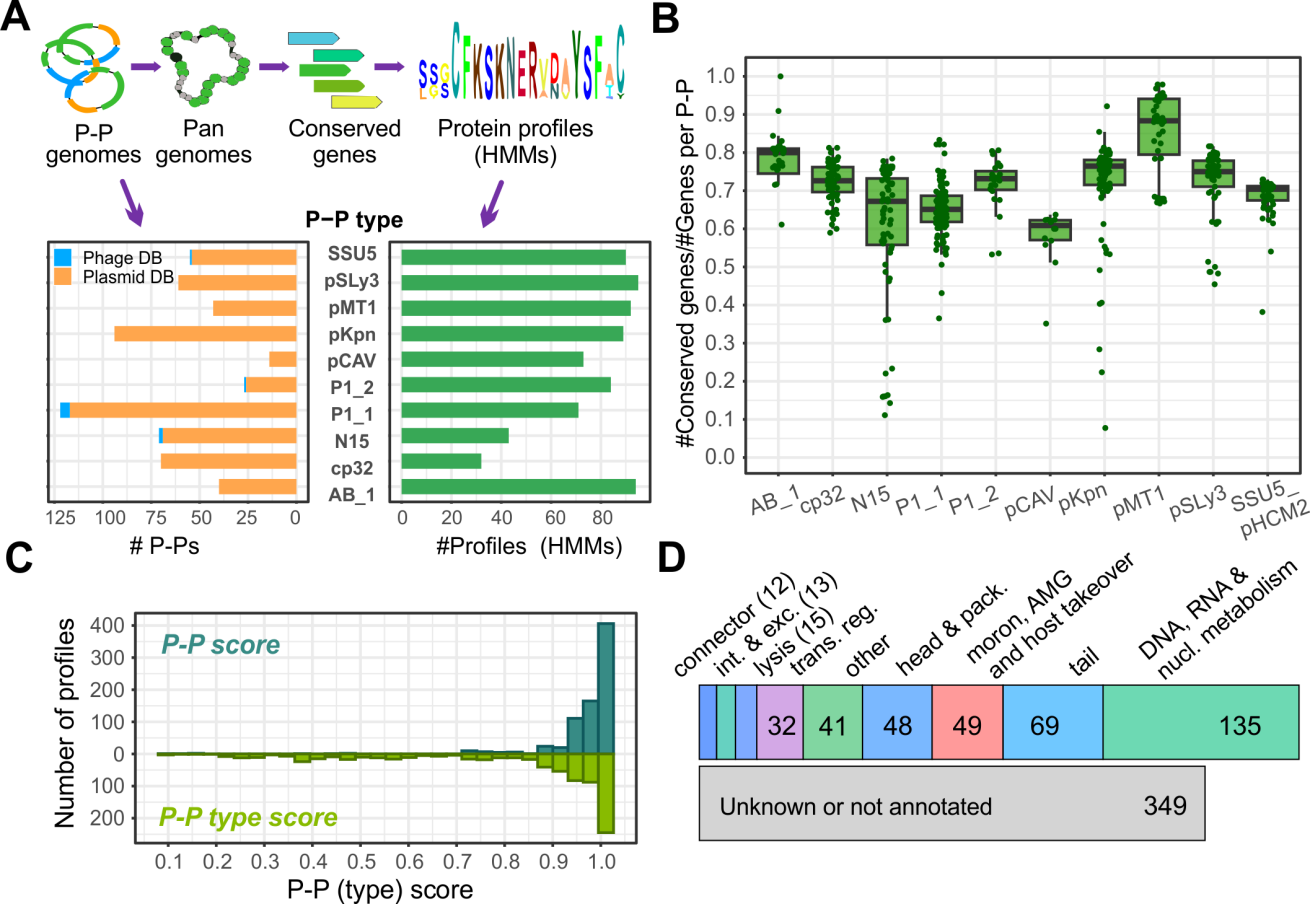
Generation and characterization of P-P protein profiles. (**A**) P-Ps from 10 curated groups (9) were selected and their pangenomes computed with PPanGGOLiN (34). Conserved sequences (classed as persistent gene families) were extracted, protein sequences were aligned with MAFFT, manually inspected and curated, and used to generate HMMs (see Methods). The number of processed P-P genomes varied per type and resulted in a total of 763 profiles. (**B**) Number of conserved genes in relation to the total number of genes per genome (across all P-P types). (**C**) P-P (dark green) and P-P-type (light green) scores were computed by counting matches of profiles against P-Ps, P-P types, phages, and plasmids (see Methods). The two scores range between 0 (unspecific) and 1 (highly specific) and differentiate P-Ps from phages and plasmids or P-P types from other P-Ps. (**D**) Representative sequences of the 763 P-P profiles were annotated using PHROGs.

From the 10 P-P groups, we extracted conserved gene families, aligned their protein sequences, and generated HMMs from the multiple sequence alignments (see Methods, Fig. 1A). The number of P-P genomes analyzed per P-P type ranged from 14 to 122, resulting in a total of 763 HMMs, with 32 HMMs for cp32 P-Ps and 95 for pSLy3 P-Ps (Fig. 1A; Table 1). We evaluated the profiles’ characteristics, including the detection range, diversity, and specificity. On average, we found that 69.1% ± 7.7% (mean of coefficient of variance = 16.7%) of genes are conserved per P-P (Fig. 1B). To assess the profile diversity, we calculated the NEFF value per profile that represents the number of effective sequences in the alignments (38, 48). While NEFF values close to 1 indicate highly similar sequences, higher values indicate more diversity. The mean NEFF across all P-P types was 1.06 ± 0.06. We found low sequence diversity (NEFF = 1.0) for pMT1 sequences that are specific for *Yersinia pestis*. This is likely because *Y. pestis* strains are oversampled and the fact that it is a recently emerged species (49). Highest diversity was observed for N15 HMMs (up to NEFF = 1.4) and cp32 HMMs (all NEFF > 1.0) (Fig. S2; Table S2).

Next, we tested the specificity of the HMMs using two scores. The first, named P-P score, was used to differentiate P-Ps from phages and plasmids, and the second, P-P-type score, was used to determine its specificity to P-P types. The two scores range from 0 (not specific) to 1 (highly specific). Most of the protein profiles (702/763) achieved P-P scores ≥0.9, indicating very high specificity (Fig. 1C). However, only 35.4% of the HMMs from pSLy3, pKpn, and SSU5_pHCM2 reached such a high type specificity (P-P-type score ≥0.9), which is in strong contrast to 76% of the other profiles (Fig. S3; Table S2). P-Ps from these three types are all closely related to SSU5 and have high genomic similarities (9), causing their profiles to confidently match P-Ps from all three types. Nonetheless, these HMMs can provide valuable information to distinguish these P-Ps from all other P-P types.

To assess functions of the profiles, we annotated representative protein sequences using PHROGs (37), protein profiles specific for replicases and partition systems (50), and the database of geNomad (19). In total, we annotated 64% (488/763) (Table S2), and essential phage (structure, lysis, and connector) and plasmid (replication and partition) functions were covered for all P-P types, albeit with varying annotation rates. In particular, while most P1_1 profiles (79%, 56/71) could be annotated, cp32 profiles were the fewest (19%, 6/32) (Fig. 1D; Fig. S4; Table S2).

In summary, our P-P profiles match on average 70% of P-P proteins, are highly specific to P-Ps (and their types), and cover essential functions of phages and plasmids. From these characteristics, we concluded that these HMMs will function as signature profiles facilitating P-P detection and typing.

### tyPPing uses patterns of conserved P-P proteins to accurately detect and type P-Ps

We used the 763 protein profiles to develop tyPPing that is a workflow for detecting and typing P-Ps. tyPPing requires a profile-to-protein comparison table (produced with the 763 protein profiles), a protein-to-genome, and a genome size table (for details, see https://github.com/EpfeiferNutri/Phage-plasmids/tree/main/tyPPing).

**Fig 2:**
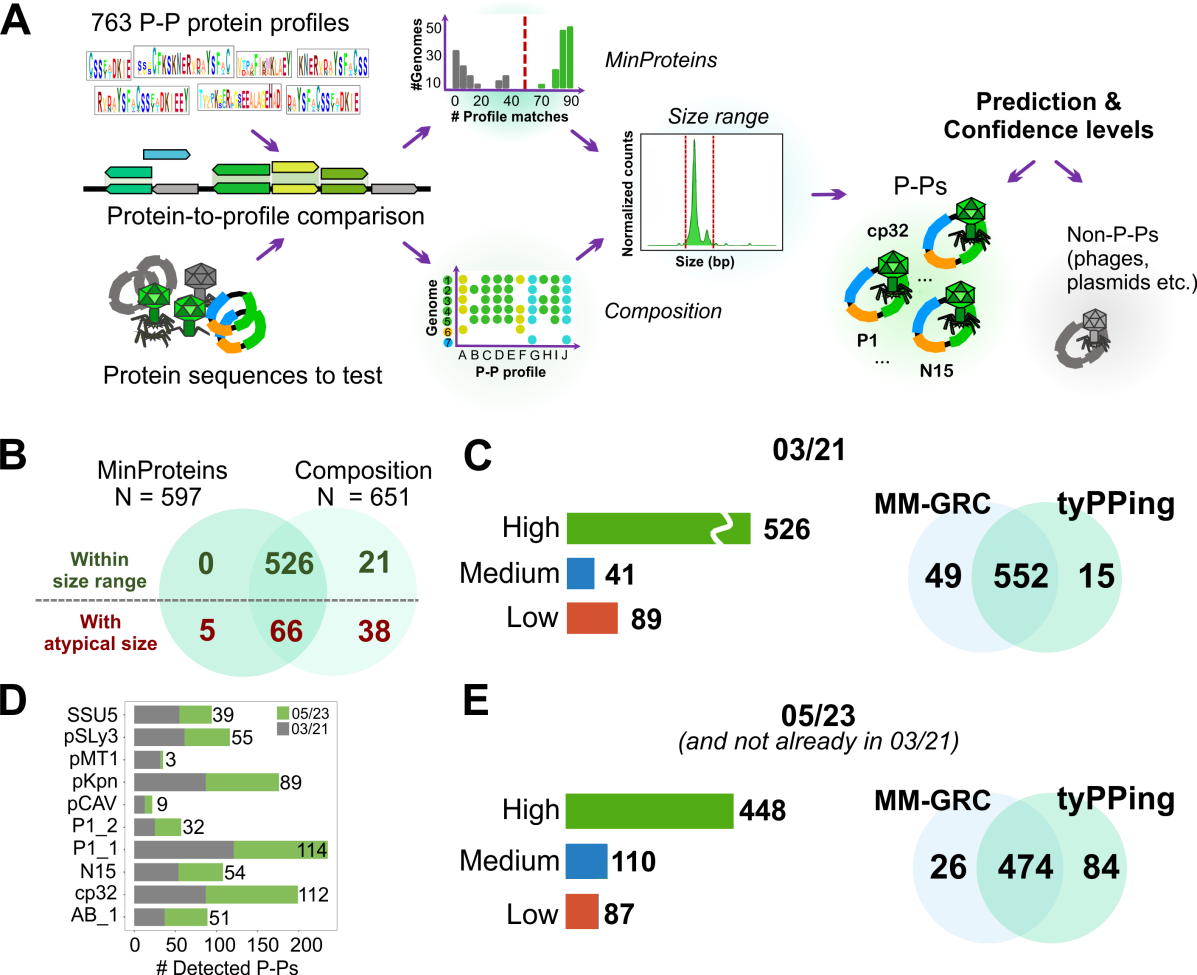
Detection and typing of P-Ps with tyPPing. (**A**) The 763 signature profiles are used to generate a protein-to-profile table by comparing them against protein sequences (encoded by the tested genetic element). This table is read by tyPPing that evaluates if matches meet the defined MinProteins, Composition, and size range thresholds for the 10 P-P types. To sequences that pass MinProteins or Composition, it assigns confidence levels that depend on the match to the size range (see Methods). (**B**) tyPPing was employed on the 03/21 data set, and hits specific for MinProteins and Composition were compared, including the number of elements fitting the type-specific size ranges. (**C**) Confidence levels of putative P-Ps in 03/21 (bar plot) and intersection of tyPPing and MM-GRC. (**D**) tyPPing was used to identify P-Ps in 05/23. P-Ps (group-wise) already detected in 03/21 (gray) and in 05/23 (green). (**E**) Confidence levels of putative P-P sequences of 05/23 (and not already in 03/21) predicted by tyPPing and comparison to MM-GRC predictions.

tyPPing first computes two parameters (Fig. 2A): (i) MinProteins and (ii) Composition. For MinProteins, tyPPing counts the number of highly conserved proteins in the target sequences and keeps only those that pass a threshold (Table 1; Fig. S5 to S6). In Composition, all proteins that align to at least 50% of the HMM sequences are considered. We observed this to be less stringent than the sequence threshold used in MinProteins (Fig. S7). Here, tyPPing keeps only target sequences if it detects protein sets similar to those found in P-Ps. To achieve this, tyPPing compares the set of detected proteins (per target sequence) to those found in P-Ps. Because perfect matches (where all proteins in a set are covered) are too restrictive and can miss genetically diverse cases, we allow for a number of missing proteins per set (Table 1; Fig. S8). The thresholds for MinProteins (minimum number of conserved proteins) and for Composition (sizes of the protein sets) are distinct for each P-P type (Fig. 2A), and we tested various parameters to identify those that enable specific and robust detection (see Methods, Fig. S5 to S8).

As a last step, tyPPing assigns confidence levels to the P-P predictions (high, medium, and low). For this, it uses matches to MinProteins, to Composition, and to size ranges of the predicted P-P type (Table 2). We determined the size ranges from curated P-P types, where we excluded elements with atypical sizes (see Methods, Fig. S9). A high confidence is assigned to P-Ps that pass MinProteins, Composition, and fit the size range of the predicted P-P type. Medium confidence cases are identified by MinProteins and/or Composition and match the size range with up to 10% variation. A low-confidence case is detected by MinProteins and/or Composition and has a substantially shorter or longer genome size. We considered sequences with lengths that were either too short (potentially from degradation) or too long (potentially from recombination events or sequencing errors, see Fig. S10) to be unlikely P-Ps with functional lytic cycles of phages. This is because they may lack essential genes (too short) or are impaired in packaging the whole genome into the virion (too long). Nonetheless, some of them may represent notable cases of interest (such as defective P-Ps). This is why tyPPing reports these low-confidence cases, and we suggest a closer inspection if they are of particular interest.

We first tested tyPPing on a database containing over 25,000 complete phages and plasmids (hereafter referred to as “03/21”). A total of 656 elements were detected by MinProteins (*n* = 597) and Composition (*n* = 651) with 592 overlapping cases (Fig. 2B). Most elements (79%) were classified as high confidence, 7% as medium, and 14% (*n* = 89) as low (Fig. 2C). Among the low-confidence elements, seven were identified as too short, while 82 exceeded the expected size ranges. We observed that tyPPing detects sequences that are too long more often than sequences being too short. This is because too-short sequences do not typically pass tyPPing’s cutoffs (MinProteins and Composition) and are thus not reported. We also observed that too-long sequences often harbor additional sequences with high similarity to plasmids. This suggests that recombination events (between P-P and plasmid) caused these increased sizes (examples shown in Fig. S10).

As a next step, we compared tyPPing with our first approach to detect P-Ps, which we will refer to as multi-model gene repertoire clustering, or short “MM-GRC” (9). In this method, we use phage and plasmid HMMs for functional annotation, random forest models for P-P detection, and employ the gene repertoire relatedness (wGRR) to cluster and type P-Ps (see Methods). The workflow of MM-GRC differs for replicons initially classed as phage or plasmid sequences. Specifically, phage sequences are screened by plasmid-specific HMMs for plasmid-related functions. Plasmids are analyzed by phage-specific HMMs and then classed by random forest predictors (9). We first compared tyPPing’s predictions with those of MM-GRC using the 03/21 data set. tyPPing detected 567 putative P-Ps, and MM-GRC detected 601 (in total, 616 putative P-Ps) (Fig. 2C). MM-GRC uniquely detected 49 cases, with tyPPing classifying 43 of those with low confidence (Table S1). The remaining six did not match the threshold of MinProteins or Composition. tyPPing uniquely detected 15 cases, all with medium confidence, of which 13 were assigned to cp32 (examples in Fig. S11) and two to P1_1 (NZ_CP010146 and NZ_LR883966). MM-GRC does not use cp32-specific phage profiles but relies on the clustering to detect cp32 P-Ps. We noted that the threshold for clustering excluded the detection of these elements. The two P1-related cases (NZ_CP010146 and NZ_LR883966) were only detected by Composition. We found that these two cases have most of the conserved P1_1 proteins but lack proteins important for virion structure and assembly (Fig. S12). They also encode origins of transfer (*oriT*) sequences, a hallmark of elements mobilizable by conjugation. We therefore classify them not as P-Ps but as P1-like plasmids that probably evolved from P1-like P-Ps (18).

Finally, we tested tyPPing on a more recent genome database generated in May 2023 (“05/23”). This database has 77% more plasmid sequences in comparison to 03/21 containing 38,051 elements labeled as plasmids. We focused on plasmids since the vast majority of P-Ps (90.2% in 03/21, Table S1) were detected in previous plasmid databases. While tyPPing required 8 min to process 05/23 (and additional 104 min for the protein-to-profile comparison), MM-GRC needed 25 h (using the same machines, 25 CPUs). tyPPing identified a total of 558 P-Ps that are not already present in 03/21 with at least medium confidence (increase by 104% in comparison to 03/21). Of these, 448 P-Ps were predicted with high and 110 with medium confidence (Fig. 2D and E). Other 87 cases were predicted with low confidence, of which all exceeded the size ranges. Among the detected elements, 84 were unique to tyPPing and 26 to MM-GRC (Fig. 2F). Of the 84 tyPPing-specific cases, 83 were assigned to cp32, underlining again its high sensitivity toward cp32 P-Ps. The remaining one was predicted to be a P1_2 P-P (medium). This element encodes conserved P1_2 genes and lacks many essential phage genes (Fig. S13), suggesting that it is not a P-P but a P1-like plasmid. Of the 26 cases distinct for MM-GRC, 21 have atypical sizes (too short) or were classed as low by tyPPing. Three of the other cases matched the protein profiles of AB_1 but did not pass thresholds of MinProteins or Composition. We suggest they are defective P-Ps since they lack many conserved AB_1 proteins (Fig. S14). The remaining two, detected only by MM-GRC, are SSU5-like P-Ps, one of *Buttiauxella* (NZ_CP110081) and one of *E. coli* PI6 (NZ_CP074043) (Fig. S15). These sequences did not pass MinProteins and Composition thresholds.

In summary, tyPPing efficiently detects P-Ps of different types and provides confidence scores for the predictions. In comparison to MM-GRC, it reaches >99% sensitivity and >99% precision, and despite a few misclassifications (in total three false positives in 03/21 and 05/23), it is capable of curating P-P data sets by reporting low-confidence cases.

### P-P detection advances with distinct classification strategies

**Fig 3:**
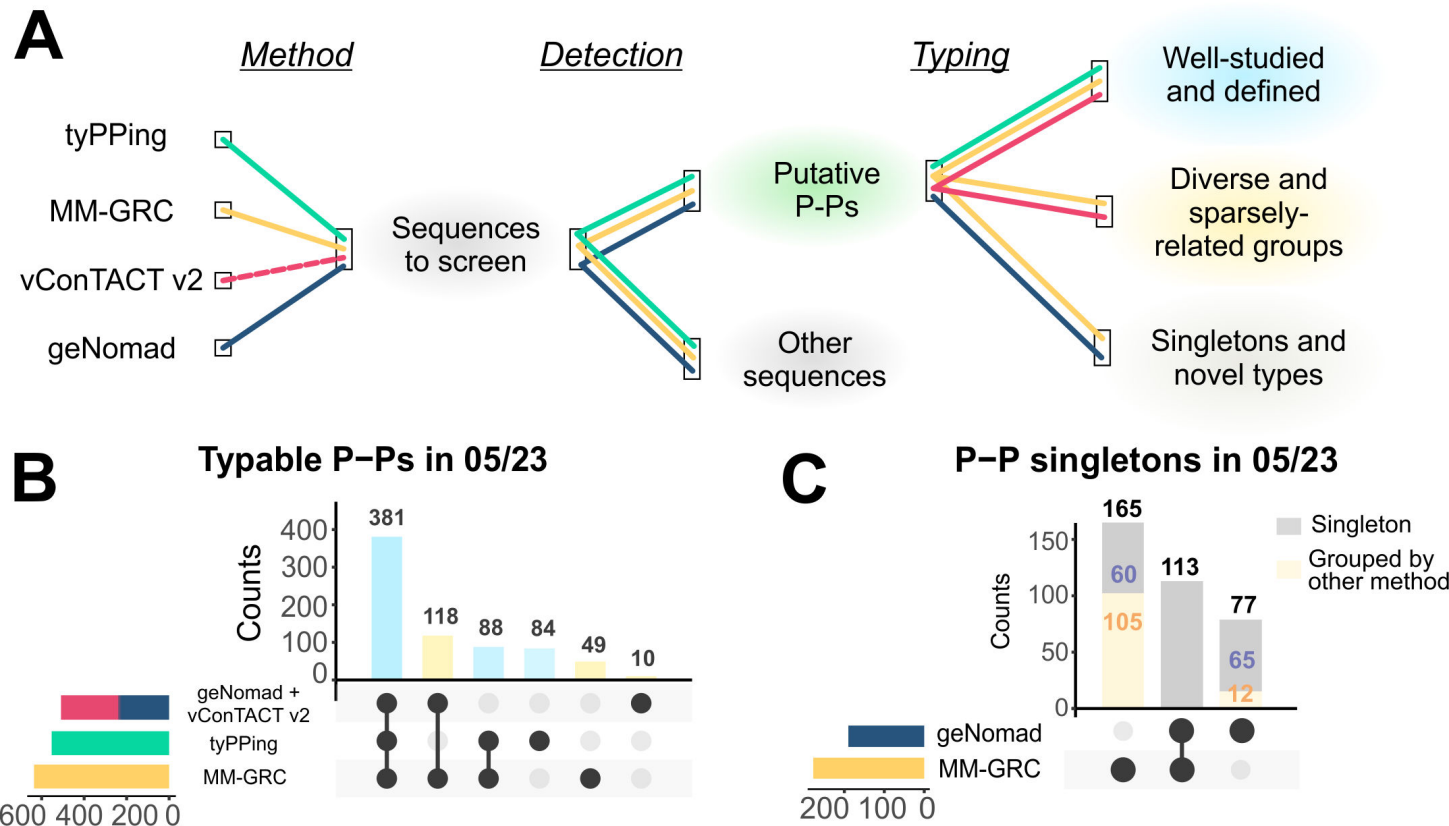
Detection of P-Ps using distinct genomics approaches. (**A**) P-Ps are categorized in well-defined (blue), sparsely related groups (yellow), and singletons (gray). Specific methods such as tyPPing, vConTACT v2, geNomad, and MM-GRC were compared in their performances to predict and type P-Ps in 05/23. (**B**) Counts of P-Ps, well-defined types (blue) and sparsely related groups (yellow), identified by tyPPing, MM-GRC, and geNomad combined with vConTACT v2. P-Ps detected by any method as singletons were excluded. (**C**) Counts of P-P singletons detected by MM-GRC and geNomad (gray bars). Some P-Ps were detected as singletons but were grouped by the other methods (yellow bar, orange numbers).

In a previous work, we separated well-related P-Ps, which cluster with experimentally characterized examples, from sparsely related groups and distinct individuals (singletons) (9). The well-related types are detected by tyPPing, whereas MM-GRC also identifies the other types (Fig. 3A). We wondered if current classification tools, such as geNomad and vConTACT v2, could be used to improve P-P detection specifically, focusing on aspects like robustness, P-P typing, and the potential to spot novel P-Ps.

We first tested geNomad, which types sequences either as phages, as integrated prophages, or as plasmids (19). We analyzed all plasmids of 05/23 and considered elements classed as phage-positives as P-Ps. We excluded those identified as integrated prophages as, per definition, they are not P-Ps. In total, geNomad required 6 h and 17 min (using 25 CPUs) for the 38,051 sequences and detected 1,714 putative P-Ps, of which 807 were not already present in 03/21 (Table S1). Since geNomad does not group sequences, it cannot type P-Ps. Notably, geNomad predicted 19.5% to 30.8% of the P-Ps detected by MM-GRC or tyPPing, respectively, as plasmids and/or integrated prophages (Fig. S16A).

Next, we tested vConTACT v2, a guilt-by-association classifier (25). vConTACT v2 clusters sequences by computing gene-sharing networks with a reference data set. We used the 1,416 P-Ps of 03/21 (18) as the references, and sequences that co-clustered with them were considered putative P-Ps. In total, vConTACT v2 required 168 h and 41 min for 05/23 (with 25 CPUs) and clustered 1480 cases with the P-Ps (Fig. S16B). We inspected these cases and noted that 759 (51.3%) are plasmids that clustered with a few putative P-Ps having similar genes. We tested a stricter clustering analysis by excluding overlapping and weakly associated cases (see Methods), which, however, did not resolve the plasmid and P-P clusters. This suggests that if solely used, vConTACT v2 detects a high number of plasmids as P-Ps (making >50% false positives).

To mitigate limitations of geNomad (inability to type P-Ps) and vConTACT v2 (high number of false positives), we devised a combined approach. For this, we first used geNomad to separate plasmids and putative P-Ps and then applied vConTACT v2 for the P-P typing. This approach detected 381 P-Ps of the well-related types (68% of the tyPPing-positives) and assigned them consistently as tyPPing (Fig. 3B). A total of 172 P-Ps identified by both tyPPing and MM-GRC were not detected and were classed either as outliers (by vConTACT v2) or not as phages (by geNomad). Moreover, geNomad/vConTACT v2 assigned 118 P-Ps to the sparsely related P-P groups consistently as MM-GRC. While MM-GRC placed additional 49 P-Ps, geNomad/vConTACT v2 clustered 10 cases into distinct groups.

Lastly, we tested the methods’ ability and specificity in the detection of P-Ps that do not cluster with other P-Ps, which we will refer to as singletons. These P-Ps may represent novel P-P species or types, making them intriguing candidates for understanding P-P diversity and phylogeny. vConTACT v2 and tyPPing do not detect them as vConTACT v2 relies on a reference data set, while tyPPing detects and types simultaneously. Thus, we considered P-Ps that were predicted by MM-GRC and geNomad and counted them as singletons if they did not group with any other P-P. By this, we detected a total of 355 putative singletons, of which, however, some P-Ps had conflicting classifications. While one method detected them as singletons, another one clustered them with other P-Ps. For instance, MM-GRC identified 165 P-P singletons, but 105 clustered with other P-Ps by the other methods. Conversely, of the 77 P-P singletons specific for geNomad, MM-GRC grouped 12 of those with other P-Ps (Fig. 3C). In summary, tyPPing outperforms any other method in terms of efficiency, speed, and sensitivity to detect well-related P-P types. MM-GRC and geNomad/vConTACT v2 are effective to also spot P-P singletons and weakly connected P-P clusters.

### tyPPing detects functional P-Ps in drafted genomes using a cumulative counting mode

In a final assessment, we wondered if we can use tyPPing to effectively screen unfinished (draft) bacterial genomes for P-Ps. For this, we modified tyPPing and changed its counting system (of conserved P-P proteins) to a cumulative mode. This enabled P-P detection even if the conserved proteins are split across multiple contigs. However, with this setting, tyPPing cannot distinguish multiple P-Ps of the same type (e.g., two P1_1 P-Ps) within a single genome as counts of conserved proteins would be masked. We assume such events are rare, as same-type P-Ps are likely incompatible (as widely reported for plasmids [22]). Importantly, P-Ps of different types (e.g., P1_1 and N15) are still detected. We then tested if matches to plasmids and to chromosomal regions (e.g., to integrative prophages) could cause, if collectively counted, a false-positive P-P prediction (see Methods). For this, we screened all bacterial assemblies that contain plasmids and aggregated the matches (Fig. S17). We never observed that hits to plasmids combined with hits to chromosomes led to a positive P-P prediction. We did, however, observe seven regions solely within bacterial chromosomes that are related to P-Ps with 5 to P1_1, 1 to P1_2, and 1 to pKpn (Table S3). Furthermore, we noted that 12–14 distinct N15 profiles match 460 chromosomal sequences of *Klebsiella* species (Fig. S17). These regions are described as conserved structural gene cassettes (40) and are not typically detected in extrachromosomal sequences. These cases could lead to false positives if tyPPing is used on draft genomes in which chromosomal sequences are assembled into too many small contigs. To avoid this, we increased the N15 MinProteins threshold from 7 to 24 (Fig. S17) for cases that do not match Composition.

We then tested this version on a collection of draft genomes (short-read assemblies) from carbapenem-resistant *Enterobacteriales* species. We focused on a subset for which we also had corresponding long-read assemblies. We detected all P-Ps that we found in previous work (that also encode ARGs) (9) with one medium- and eight high-confidence cases (Table 3) (14). Moreover, we screened three further strains and detected two P1-like P-Ps in two *Escherichia coli* isolates, 170D8 (P1_1) and 174J8 (P1_2), and one N15-like P-P in *Enterobacter cloacae* 204G7. We observed that tyPPing’s predictions varied with the quality of the assemblies. Specifically, if sequences were just partly or not assembled, then even the collective matches may not meet criteria of MinProteins or Composition, and thus tyPPing may miss these cases (Table 3, detailed in Table S4).

**Fig 4:**
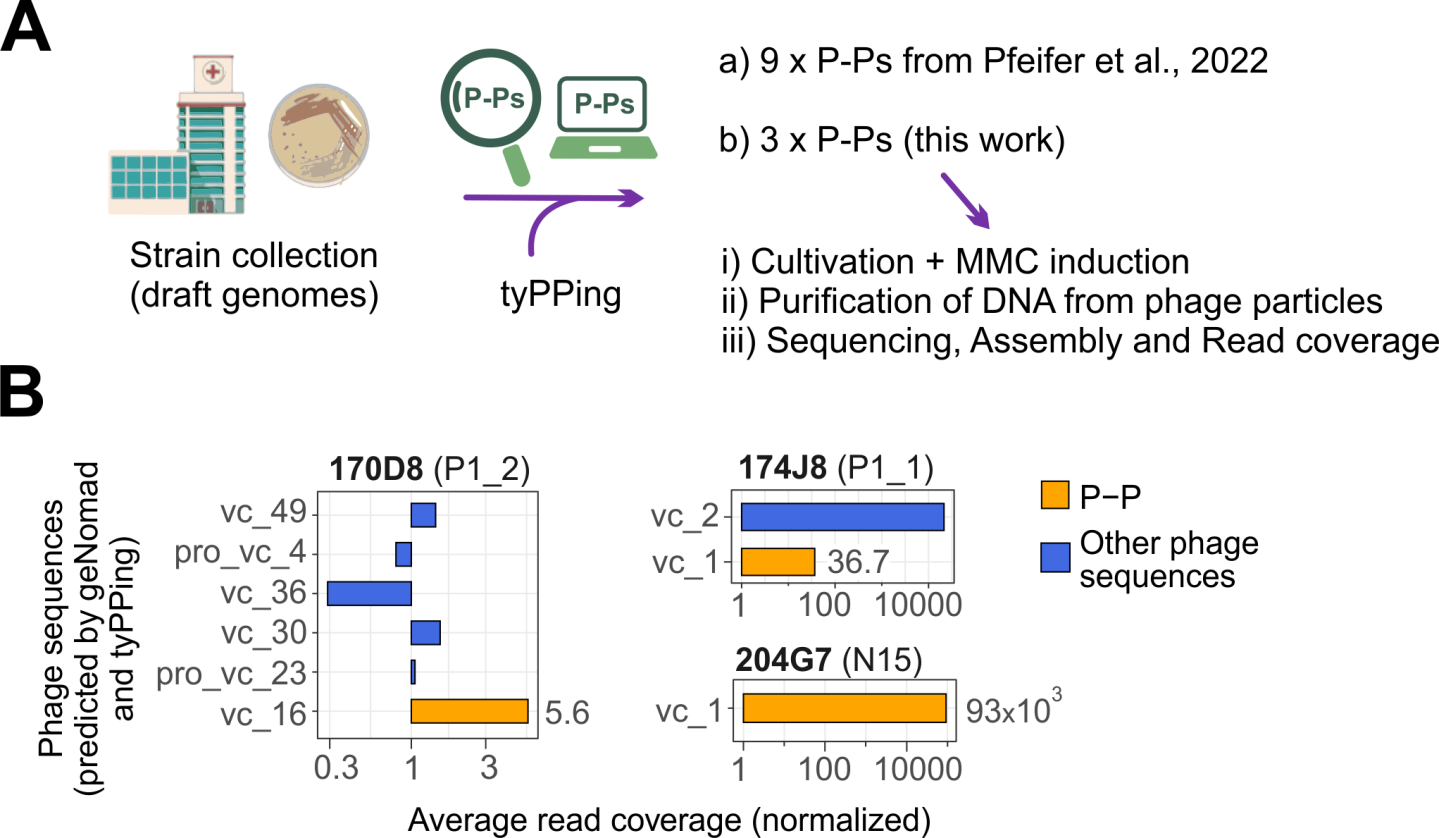
Testing the functionality of P-Ps predicted in draft genomes (by tyPPing). (**A**) We modified tyPPing to screen a collection of draft genomes (of carbapenem-resistant species). We detected all 9 P-Ps that we found in previous work (14) and detected 3 further P-Ps in three other strains (174J8, 170D8, and 204G7). We tested if these three P-Ps are responsive to MMC and packaged into virions upon treatment (see Methods). DNA was extracted from virions, used to conduct a hybrid assembly, and mapped on assemblies to compute the read coverages. (**B**) The hybrid assembly resulted in complete P-P genomes (orange) and other phage sequences (blue, predicted by geNomad). We mapped the reads from the MMC induction experiment on all assembled P-P and phage sequences, computed the read coverages and normalized these values (of P-P and phage sequences) to the level of the host chromosome.

We then wondered if the three P-Ps in the three strains 170D8, 174J8, and 204G7 are functional phages and tested if they can be induced with DNA damage (causing a switch into the lytic cycle). For this, we cultivated the strains in the presence of MMC, purified phage particles from the supernatants, digested unprotected nucleic acids (mostly from the bacterial host), and extracted DNA from virions (see Methods, Fig. 4A). We then sequenced the extracted DNA by short reads and verified the presence and relative quantity of P-P DNA. For this, we first used the short reads to improve the quality of P-P assemblies by combining them with the long reads (see Methods). This resulted in complete, closed genomes of the P-Ps (Fig. S18) and also assembled other phage-related sequences (predicted by geNomad). tyPPing assigned a high confidence to these P-Ps (Table 3). Then, we computed the average read coverages of these assemblies, the ones of the host DNA (not digested, background signal), and normalized these values (signal of assemblies to background signal). We found that all P-Ps exhibited considerably higher coverages than the host’s level, with 36.7-fold for P1_1 (in 174J8), 5.6-fold for P1_2 (in 170D8), and a 93,000-fold for the N15-like P-P (in 204G7) (Fig. 4B). In addition, in 174J8, we detected a 43-kb phage (assigned as vc_2 by geNomad) alongside the P1_1 P-P. This phage is related to HK629 (NC_019711, lambda-like), exhibited high coverage (>10,000 fold), and is a residential prophage that was triggered by MMC.

Taken together, our findings demonstrate that tyPPing effectively detects P-Ps in draft genomes of short- and long-read sequences. While prediction accuracy depends on the assembly quality, tyPPing identifies high-confidence P-Ps even when the genomes are split across multiple contigs. The MMC induction experiments showed that the P-Ps are functional and packaged into virions, suggesting them to be active as phages.

## DISCUSSION

P-Ps are widespread in bacteria, exhibit a high diversity, and have distinct characteristics (in terms of gene repertoires, hosts, morphologies, and sizes) (9). By having genes similar to those of phages and plasmids, accurate P-P detection is a major challenge in microbial genomics. Phages and plasmids recombine, which is promoted by P-Ps (18) and conclusively generates a spectrum of hybrid or chimeric sequences. These events can cause multiple variations of co-integrates, such as fusions between phages and plasmids, or any combination of P-Ps with other P-Ps, plasmids, or phages. While these products retain lifecycles of plasmids (replication and transmission over conjugation termed conduction [51, 52]), they are likely impaired in completing a full lytic phage cycle. This is because recombination can either inactivate essential lytic phage genes or result in genome sizes that prevent complete packaging. For example, while P1 tolerates a size increase up to 10% (of its 93 kb genome [7]), packaging fails at larger sizes (53). Moreover, P-Ps themselves may evolve to plasmids by becoming defective phages, through gene exchanges and the accumulation of loss-of-function mutations. For instance, we found many plasmids that are related to P1 that lack some essential phage genes but encode sequences that render them as transmissible by conjugation (such as relaxase genes and *oriT*) (18). If these P-P-like plasmids emerged recently, they have many genes similar to those found in P-Ps, phages, and plasmids, which makes it difficult to distinguish them from true P-Ps or plasmids. This ultimately complicates accurate P-P detection.

We created a refined and calibrated method for detecting P-Ps that also considers their specific types: tyPPing. We tested tyPPing exhaustively on various databases, where it consistently demonstrated effective detection of P-Ps, also in comparison to other methods. For instance, tyPPing detected 13 P-Ps (cp32 in 03/21) that were missed by our previous approach (MM-GRC). MM-GRC was initially designed to detect P-Ps, but it exhibits limitations. It uses (random forest) models that were trained to specifically separate P-Ps from other plasmids by relying on the detection of phage features. Phage functions of cp32 genes were just recently described (33), explaining why these sequences were not detected (by MM-GRC). Even after manually including cp32 P-Ps, the clustering of MM-GRC still excluded the aforementioned 13 cp32 P-Ps due to strict cutoffs (that are necessary to avoid false positives such as P-P-like plasmids). Moreover, MM-GRC detects putative P-Ps that tyPPing had classed with low confidence (14.8% of well-related P-Ps in 03/21). These sequences, either too long or too short, are puzzling as they appear to be co-integrates, shorter variants, or sequences undergoing degradation. We speculate that some of these variants might be examples of P-Ps becoming plasmids, as we observed in previous work that some plasmids emerge from P1 P-Ps (18). We generally suggest a manual inspection and careful consideration of low-confidence cases since, without experimental work, one cannot accurately ascertain these are functional or defective P-Ps. Lastly, MM-GRC needs to be applied separately on phage and plasmid sequences, lacks automation (requires numerous, manual steps), and is constrained by poor scalability (due to intense clustering). Nonetheless, it correctly recognized five elements that were misclassified by tyPPing.

We compared our methods, tyPPing and MM-GRC, to geNomad and vConTACT, tools that were not specifically made to detect P-Ps. geNomad efficiently detects MGEs (19), but it does not cluster them. We counted elements of a plasmid database as P-Ps if they were classed as phages by geNomad. vConTACT v2 is limited in the scalability (required >7 days to cluster >38,000 sequences of the plasmid database), and predictions strongly depend on the used references. For instance, we noted if a putative P-P (experimentally not confirmed) encodes too many plasmid-like genes, and vConTACT v2 co-clusters these cases with plasmid sequences, causing a substantial increase in false positives (>50% of detected elements). If combined (P-P detection with geNomad, typing with vConTACT v2), the grouping was consistent with those of tyPPing and MM-GRC (for well- and sparsely related P-P types, respectively). However, we noted that geNomad annotated 19%–30% of P-Ps as plasmids or integrative prophages, which was most evident for cp32 P-Ps (classed as plasmids). We speculate that geNomad was not trained to detect cp32 P-Ps as phages (as reasoned for MM-GRC).

A current drawback in microbial genomics is the lack of robust P-P databases and classifiers (11) limiting our understanding of the roles and diversity of P-Ps. tyPPing bridges this gap by detecting P-Ps in a fast, scalable, and highly accurate manner. Its detection strategy builds on specific patterns (frequency and matches to compositional sets) of conserved, type-defining proteins. A key strength of tyPPing is its ability to both detect and type P-Ps, regardless of a sequence’s prior classification (as a plasmid or a phage). This is a significant advantage over other methods, and it enabled us to adapt tyPPing to screen bacterial draft genomes for P-Ps. Specifically, by this tyPPing avoids difficulties that arise if contigs belonging to the same P-P are assigned to different categories (some parts to phage-like and others to plasmid-like). We suggest that this version also works with metagenome-assembled genomes (typically described as “MAGs”) when contigs are reliably binned. Moreover, tyPPing should be compatible with metagenomic datasets, when the single assembled contigs are of high completeness and good quality (as they represent single genetic elements). Quality and completeness scores are assessed by dedicated tools such as CheckV (54). Thus, combining tyPPing and CheckV is a promising approach to mine metagenomic data sets for P-Ps and, ultimately, create dedicated P-P databases. Furthermore, tyPPing assigns confidence levels to its predictions, which enables the detection of uncertain P-Ps. For instance, it spotted 89 low-confidence cases in our current P-P set (of 1,416 elements [9]). This feature is particularly important to generate high-quality data sets as quality, richness, and robustness of P-P databases are crucial, especially in the development of future tools that benefit from machine learning models.

We adapted tyPPing for the use on draft genomes and tested it first on bacterial assemblies by collectively counting matches against plasmids and chromosomal sequences. While the combined counts never resulted in one false-positive P-P prediction, we identified seven chromosomal regions with (partially strong) similarities to P-Ps. These regions are noteworthy (some of those we reported in previous works [18]) because at a first glance, they indicate that P-Ps can become integrative prophages. However, as P-Ps lack integrases typically associated with integrative mobile elements, they do not actively regulate steps such as integration and excision. We assume that these elements are rare (supported by their very low prevalence) and got captured in the chromosome through recombination not directed by integrases resulting in defective P-Ps.

We then tested tyPPing on draft genomes and detected three novel, high-confidence P-Ps in a collection of carbapenem-resistant strains. We noted that the prediction relies on the quality of the assemblies and requires that the sequences encoding the P-P proteins are properly assembled and detected by the HMMs. We then (tested and) confirmed that these P-Ps are functional as they are packaged into phage particles (when DNA damage is triggered), showing that tyPPing detects functional P-Ps.

Currently, tyPPing is capable of detecting 10 different (and prevalent) P-P types, which is its primary limitation. We aim to expand its detection range in future works by adding more P-P specific profiles. tyPPing uses a flexible workflow that readily supports the inclusion of further types (proven in this work by including cp32 P-Ps). New P-P types should ideally group with cases that are experimentally proven to propagate as phages and plasmids. Many potential new P-Ps have been described in diverse hosts like Mycobacteria (55), *Bacillus* (56) (including Betatectiviruses [57]), *Clostridium* (58), and *Vibrio* species (59, 60). An intriguing case is *Carjivirus communis* (the prototypic crAssphage), which is highly prevalent in the human gut, and was recently reported to be a linear P-P with a broad host range (61). Since the gut microbiome is a significant reservoir for temperate phages (62, 63) and also rich in P-Ps (64), mining gut metagenomic data sets offers a promising avenue for discovering novel P-P types.

Lastly, we placed P-Ps into three categories: well-defined types, singletons, and poorly connected clusters. The high number of P-P singletons suggests that we currently assess only a small fraction of the P-P diversity. A standardized taxonomy is needed to improve our understanding, as even for the well-defined types (with many experimentally validated examples), a comprehensive classification is missing. For instance, despite being studied for decades and with hundreds of available genomes, P1, N15, SSU5, and their closely related P-Ps lack a detailed taxonomy. At most, they are described to the genus/species level or just simply referred to as “unclassified *Caudoviricetes*.”

This taxonomic gap is even more pronounced among the poorly connected groups and singletons. While these cases are intriguing, the low number of available genomes and the lack of experimental confirmation imply that significant work is still necessary to characterize them. Consequently, we suggest that the development of a clear taxonomic classification needs to be prioritized. This would not only avoid the misclassification of P-Ps but also facilitate a better understanding of their prevalence, fate, origin, and their association with bacterial hosts.

## Data Availability

R scripts, data tables, sequence, and meta-information to reproduce figures and analysis are available on the GitHub (https://github.com/EpfeiferNutri/Phage-plasmids) and Zenodo (10.5281/zenodo.16616312) repositories. The raw sequencing reads (short reads from the MMC induction experiments and long reads from the whole-genome sequencing) generated in this study have been deposited in the European Nucleotide Archive under the project accession number PRJEB92425. Any additional information required to reanalyze the data reported in this paper is available from the corresponding author upon request.

## SUPPLEMENTAL MATERIAL

Supplemental material mbio.03000-25-s0001.pdf Fig. S1–S18.

Graphical abstract mbio.03000-25-s0002.png Summary.

Table S1 mbio.03000-25-s0003.xlsx All phages, plasmids, and phage-plasmids of the 03/21 and 05/23 data sets.

Table S2 mbio.03000-25-s0004.xlsx Meta information on the 763 HMM profiles generated from alignments of conserved P-P proteins.

Table S3 mbio.03000-25-s0005.xlsx Seven regions related to P-Ps detected by tyPPing in chromosomal regions.

Table S4 mbio.03000-25-s0006.xlsx Detailed overview of P-P contigs detected by tyPPing in draft genomes using the cumulative and non-cumulative mode.

ASM does not own the copyrights to Supplemental Material that may be linked to, or accessed through, an article. The authors have granted ASM a non-exclusive, world-wide license to publish the Supplemental Material files. Please contact the corresponding author directly for reuse.
