# Supplementary material for: Efficient detection and typing of phage-plasmids
Source: mBio. 2026 Feb 9;17(3):e03000-25. doi: 10.1128/mbio.03000-25 (PMC12977533; doi:10.1128/mbio.03000-25)
Supplement: Supplemental material — Fig. S1–S18. [file mbio.03000-25-s0001.pdf]

Supplemental file to

Efficient detection and typing of phage-plasmids.

Authors

Karina Ilchenko[1], Remy A. Bonnin[2,3,4], Eduardo PC Rocha[5], Eugen Pfeifer[1,6]

Author affiliation :

1 Université Paris-Saclay, INRAE, AgroParisTech, MICALIS, 78350 Jouy-en-Josas, France

2 Université Paris-Saclay, Inserm, CEA, Center for Immunology of Viral, Auto-immune, Hematological and Bacterial diseases » (IMVA-HB/IDMIT/UMRS1184), Fontenay-aux-Roses & Le Kremlin-Bicêtre, France

3 Bacteriology-Hygiene Unit. Bicêtre Hospital Assistance Publique-Hôpitaux de Paris. Le Kremlin-Bicêtre. France

4 Associated French National Reference Center for Antibiotic Resistance: Carbapenemase-Producing Enterobacteriaceae. Le Kremlin-Bicêtre. France

5 Institut Pasteur, Université Paris Cité, CNRS, UMR3525, Microbial Evolutionary Genomics, 75015 Paris, France

6 Université Paris-Saclay, INRAE, MetaGenoPolis, 78350 Jouy-en-Josas, France

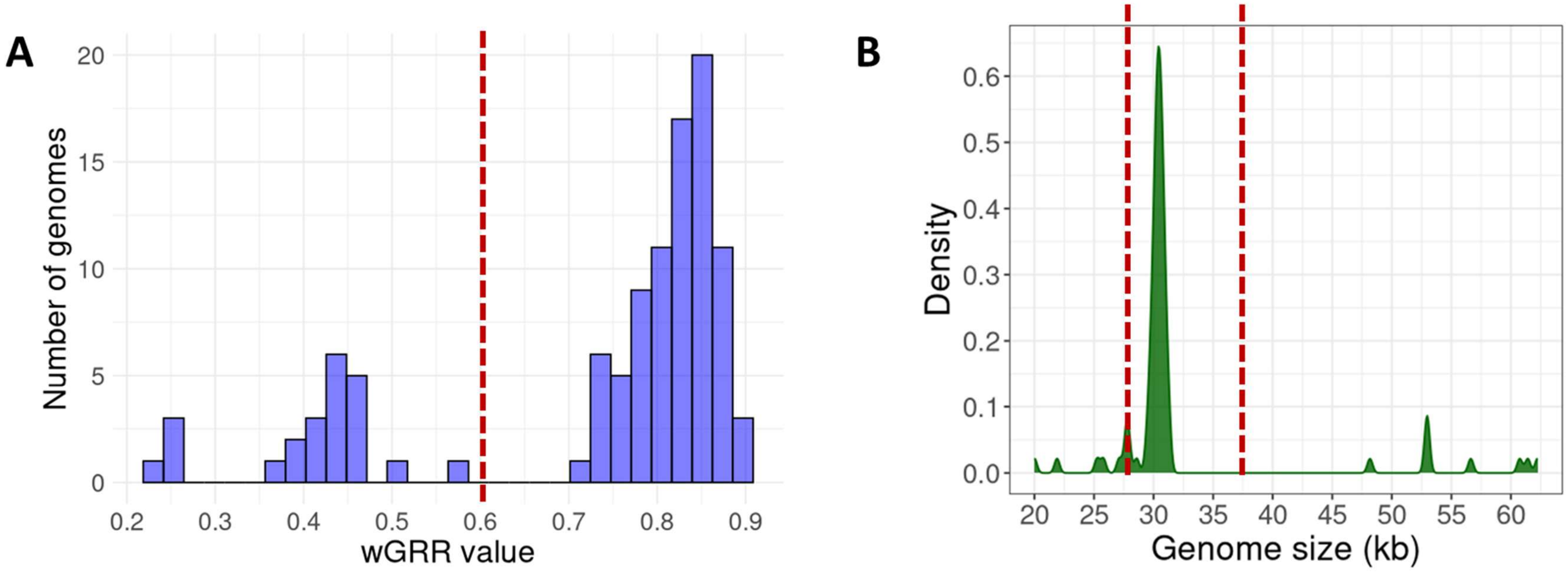

Figure S1. Defining cp32 sequences as a well-related P-P type. (A) Sequences of *Borrelia/Borreliella,* taken from 03/21, were considered as putative P-Ps if they had homologs (at least 50%) in one of the five cp32 P-Ps (1), and a wGRR >0.6. (B) Some of those elements were substantially larger or smaller than the first reported cp32 P-P (with 32 kb). To exclude co-integrates and fragmented variants, we defined elements with sizes between 27kb and 37kb (red dashed lines) as cp32 P-Ps.

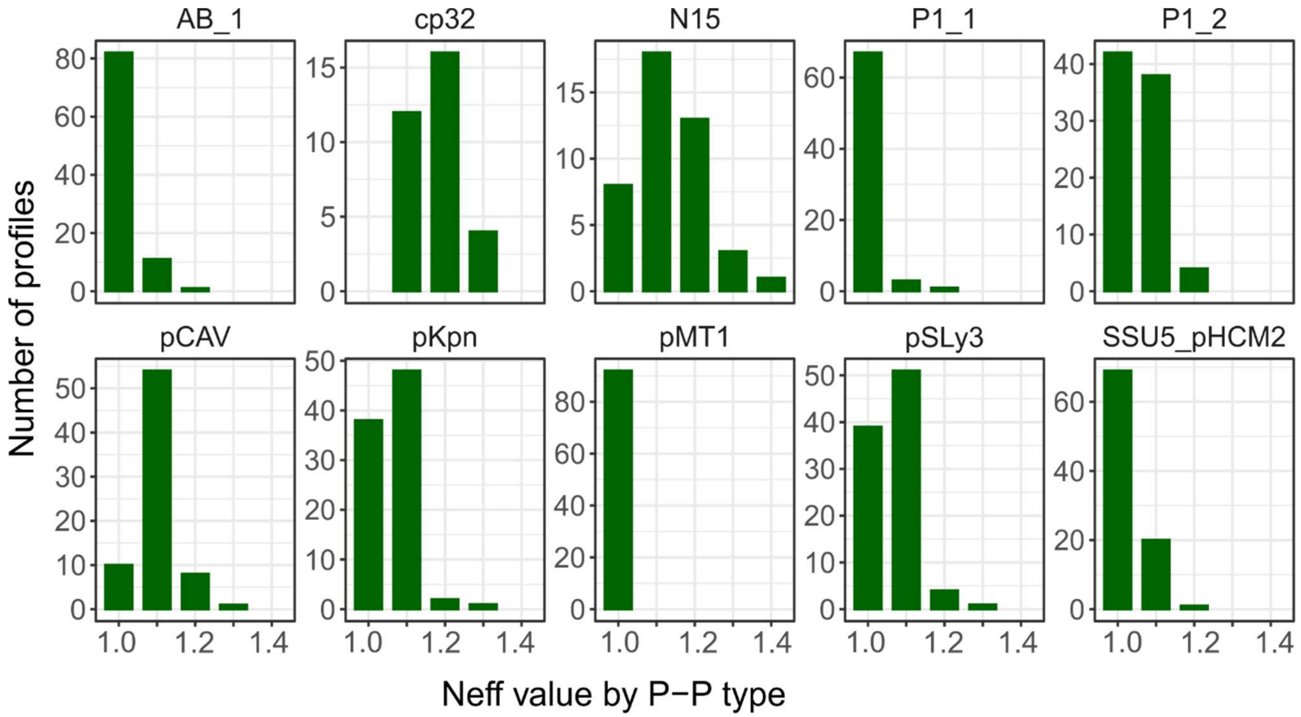

Figure S2. Neff values of P-P profiles across all well-related types. Neff values were computed using HH-suite (2), and indicate the effective sequence diversity of the multiple sequence alignment of the used protein sequences. Whereas Neff = 1 shows low diversity, higher values indicate higher diversity.

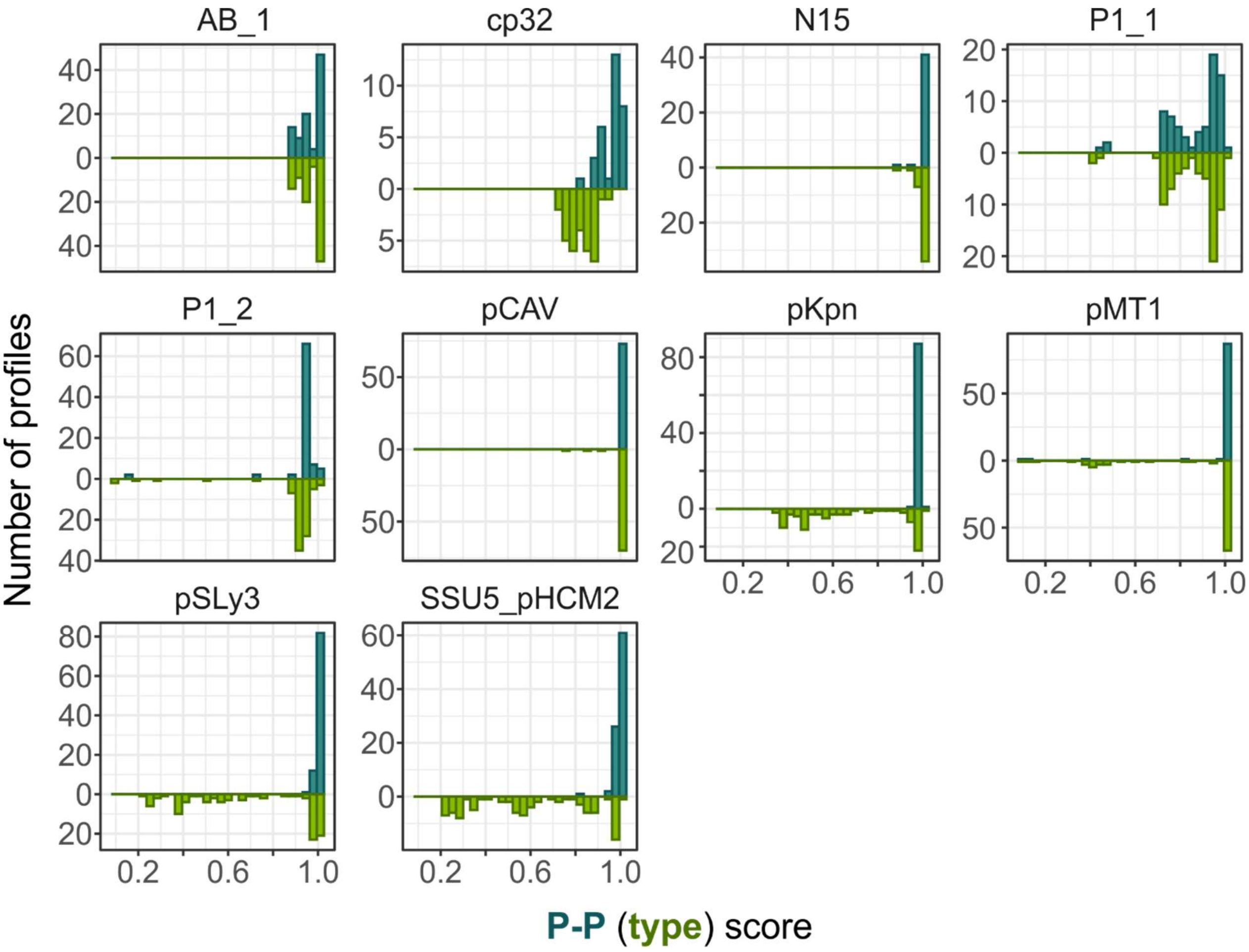

Figure S3. P-P and P-P-type score across P-P types. As in Figure 1C, but group wise.

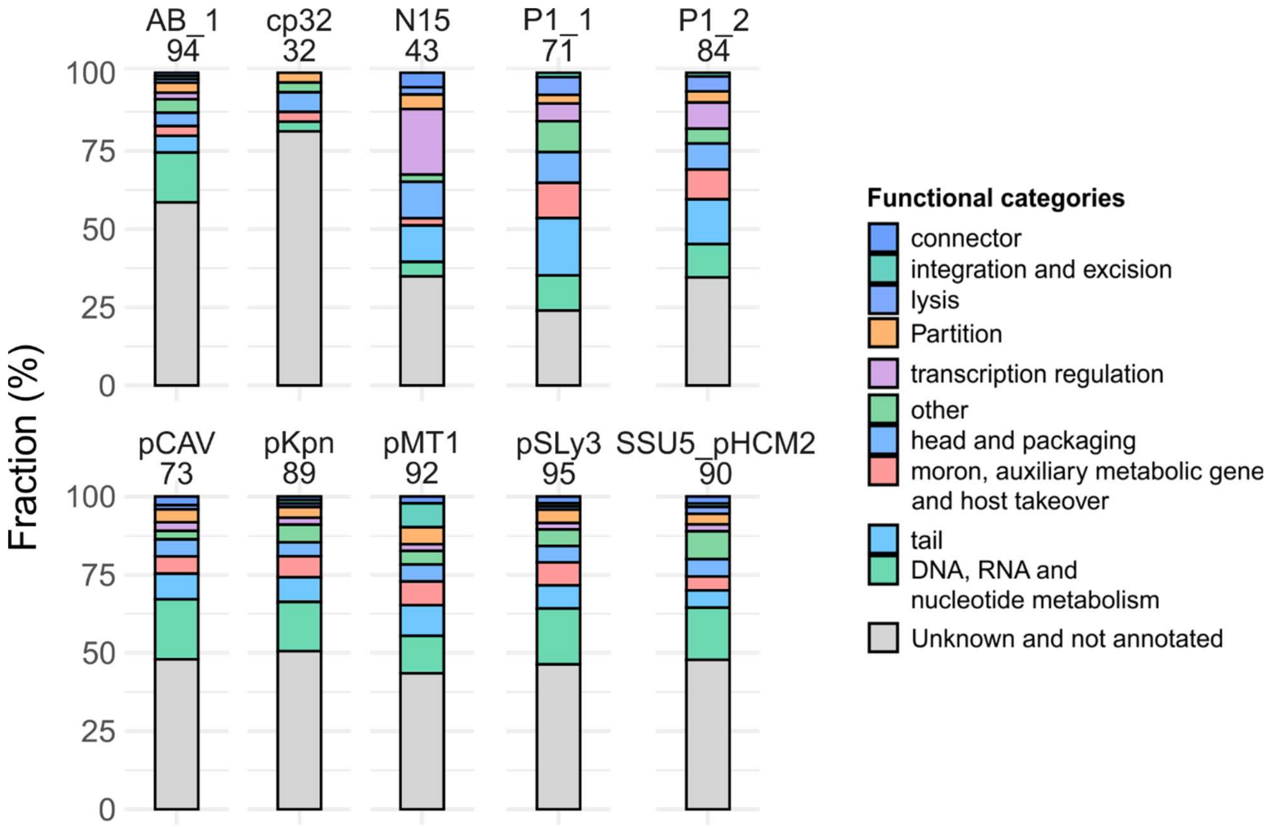

Figure S4. Annotations of the 763 P-P profiles (as in Figure 1D, but group wise)**.**

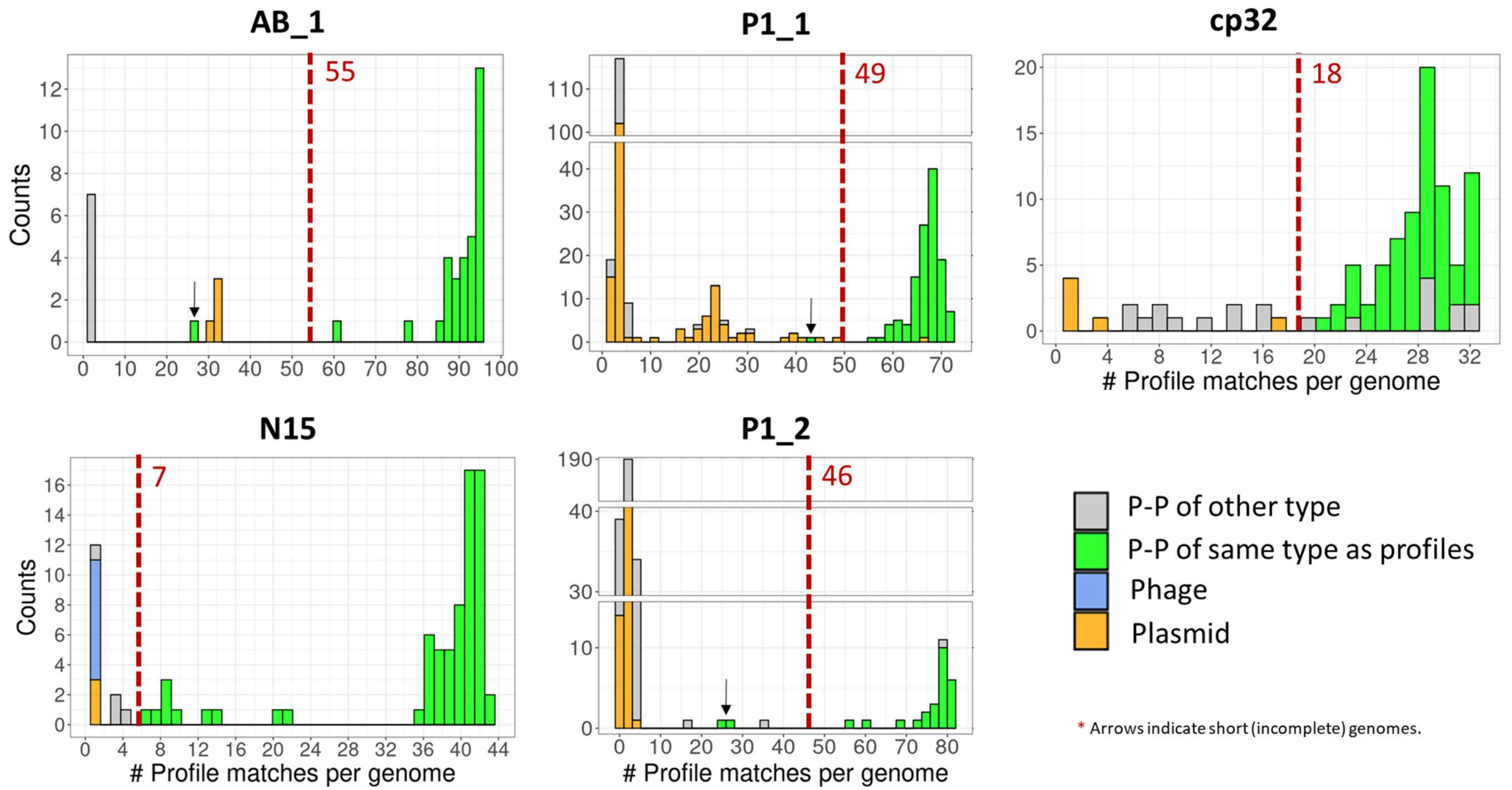

Figure S5. MinProteins thresholds. We used the 763 P-P profiles to search for minimum number of conserved proteins (for AB_1, P1_1, cp32, N15 and P1_2) that allow the differentiation between plasmid, phage and P-P (type). We counted the number of detected genomes (y-axis) and correlated it with the number of profiles that matched these genomes. Chosen cutoffs are shown in red dashed lines.

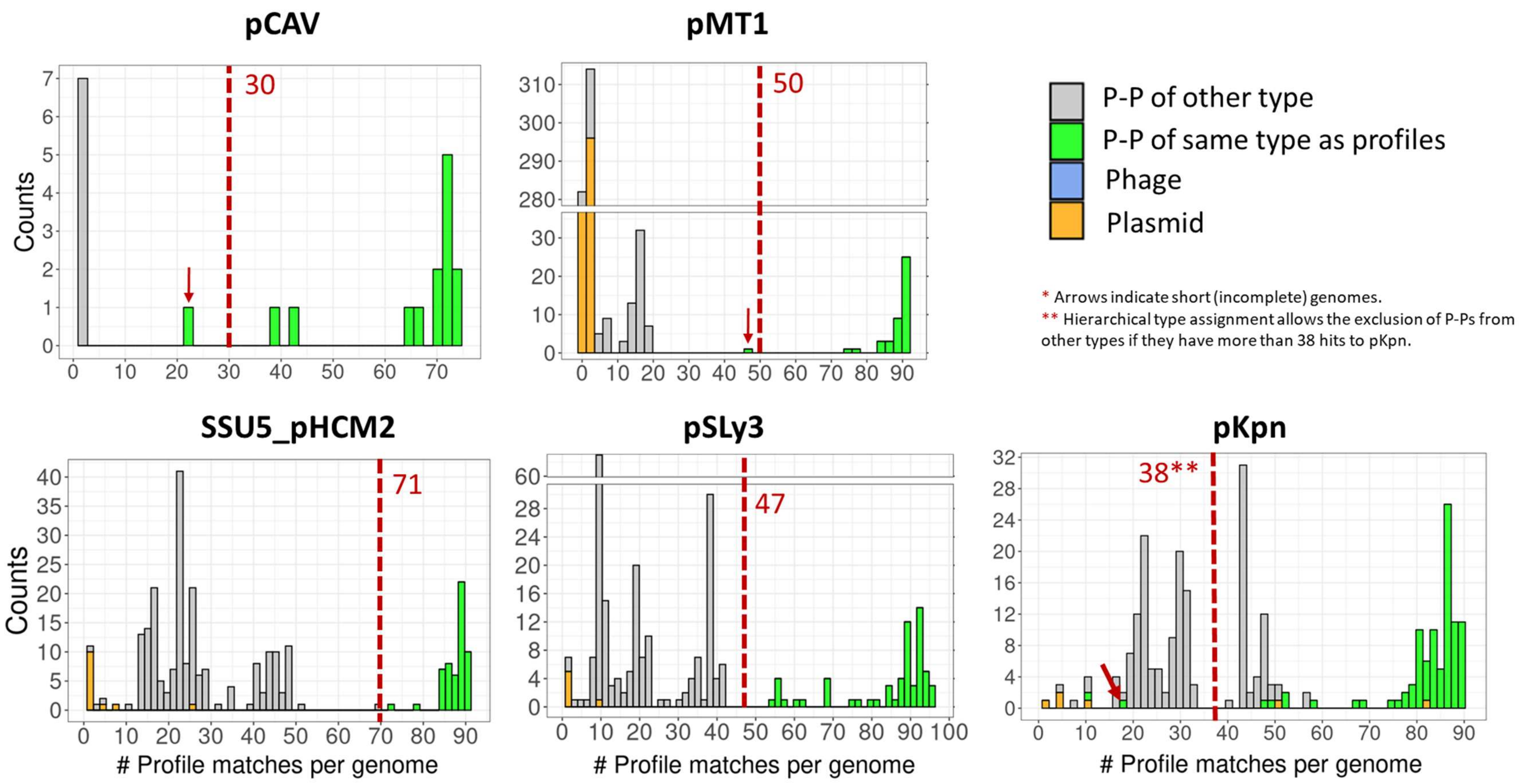

Figure S6. MinProteins thresholds. As Figure S5 but for SSU5-related P-P types.

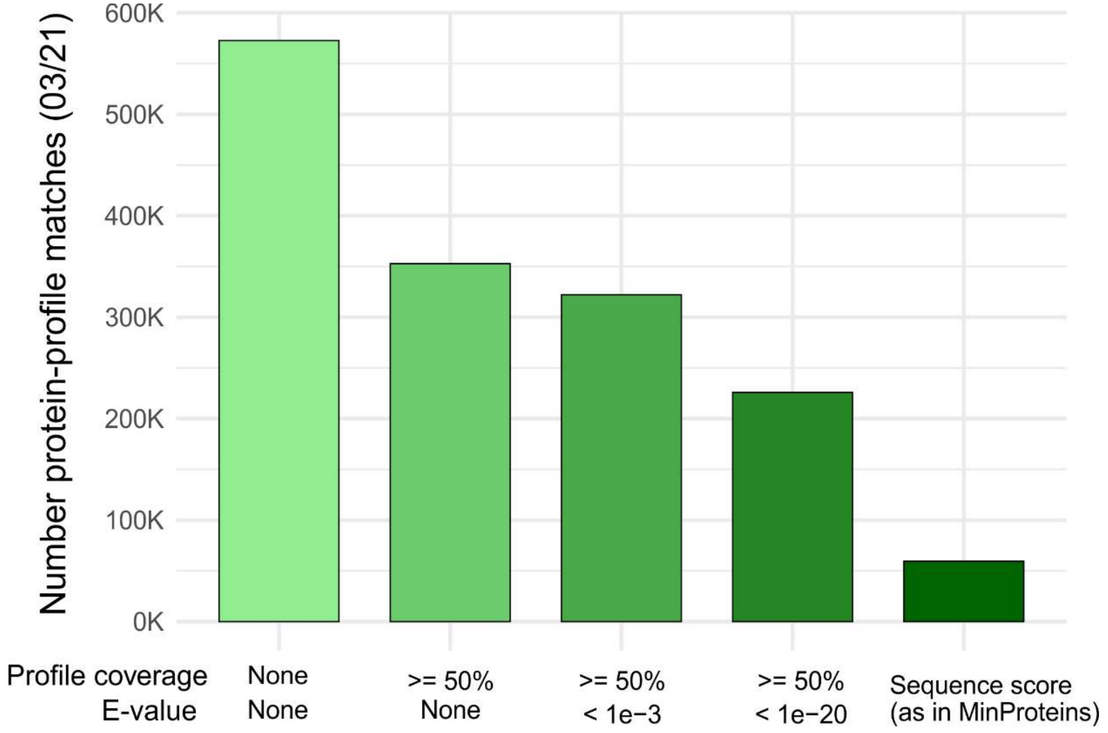

Figure S7. Thresholds for profile-to-protein hits. We evaluated several parameters, such as profile coverage and E-value, to count the number of detected proteins using the 03/21 dataset.

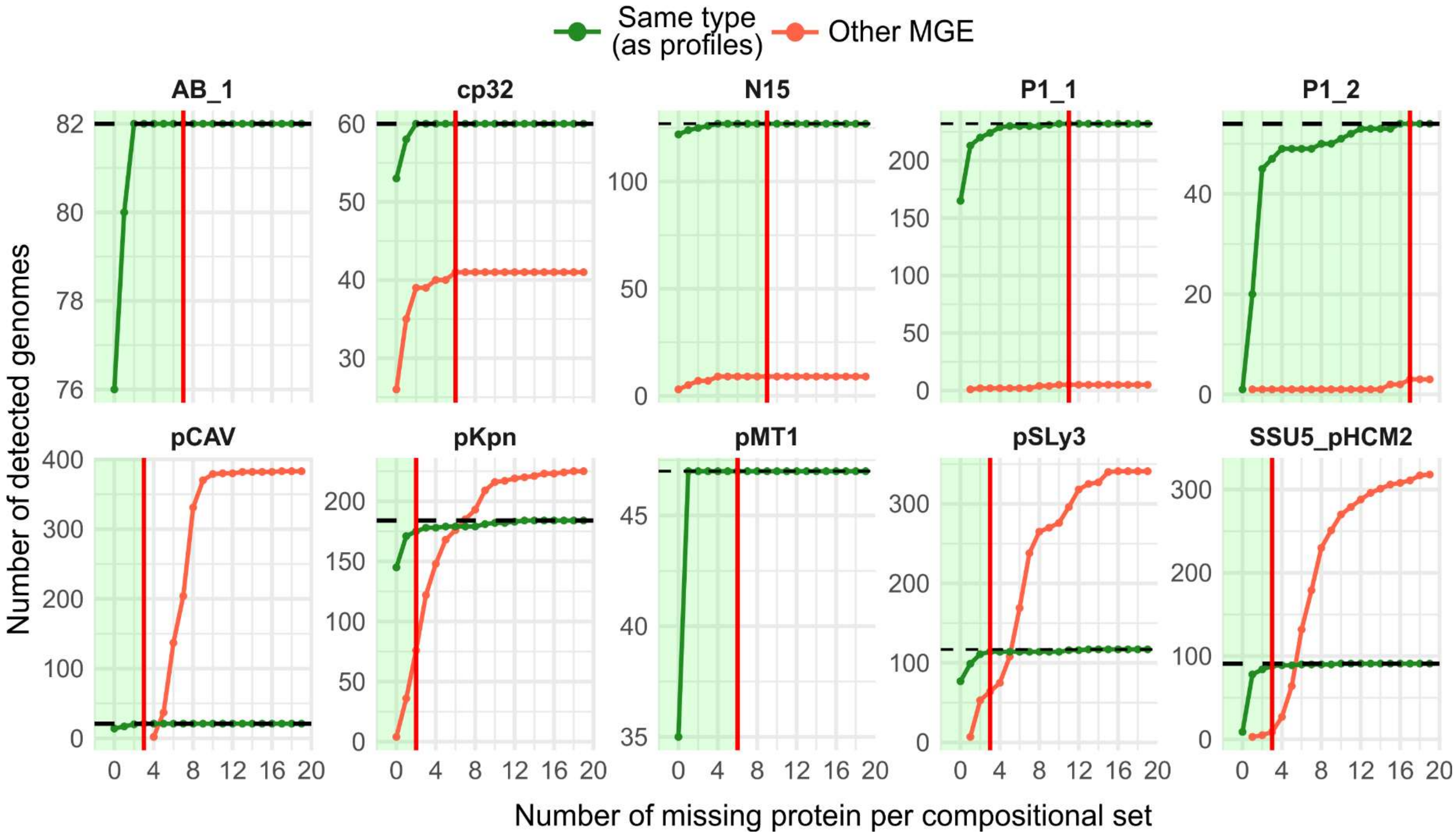

Figure S8. Thresholds for Composition. To count profile-to-protein matches, we fixed 50% coverage (Figure S7). We then varied the minimum sizes of the unique compositions by allowing distinct number of proteins not to be detected (x-axis), and tested the detection specificity, i.e. if a P-P of the same type (green curve), a different type, or a plasmid is recovered (orange curve). Red lines indicate the chosen threshold of allowed missing proteins per P-P type. Notably, to keep robust predictions, and since a high number of missing proteins has a stronger impact on compositional sets with small sizes, the maximum incompleteness was set to 75%.

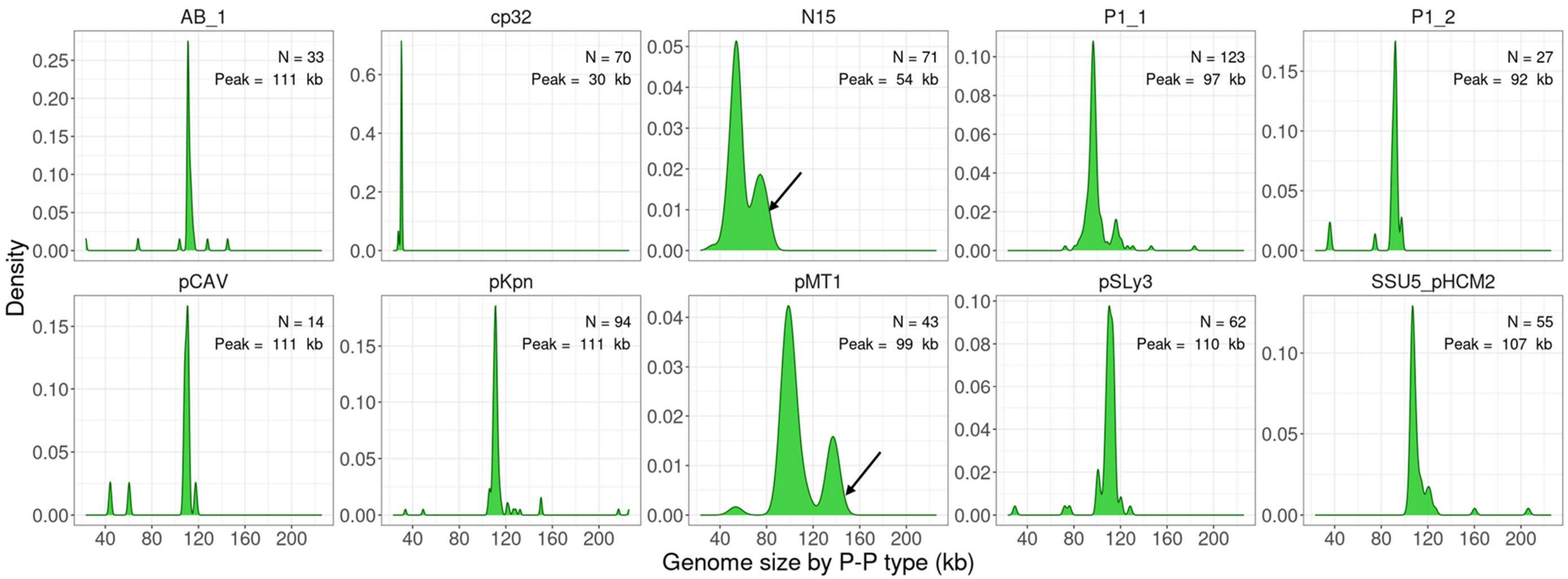

Figure S9. Size distribution of P-P genomes of different types. We first curated the P-P types by excluding sequences with atypical sizes. Specifically, genomes falling outside the major peak and sequences within a shoulder peak (indicated by the black arrows) were not considered in computing the size range. We used the mean size of the curated P-P types, ± 3x standard deviations as the ranges. We found that the unusually long N15 genomes contained long inverted repeats (see Figure S10C for examples) that are reported to be sequencing artefacts caused by wraparound reads (from sequencing linear genomes with the PacBio technology) (3). We found the second population within pMT1 P-Ps (black arrow, Inc-typed as IncFIB (4)) to be formed by >150 kb elements that are pMT1 P-Ps fused to a IncFII plasmid (4)) with conjugative genes.

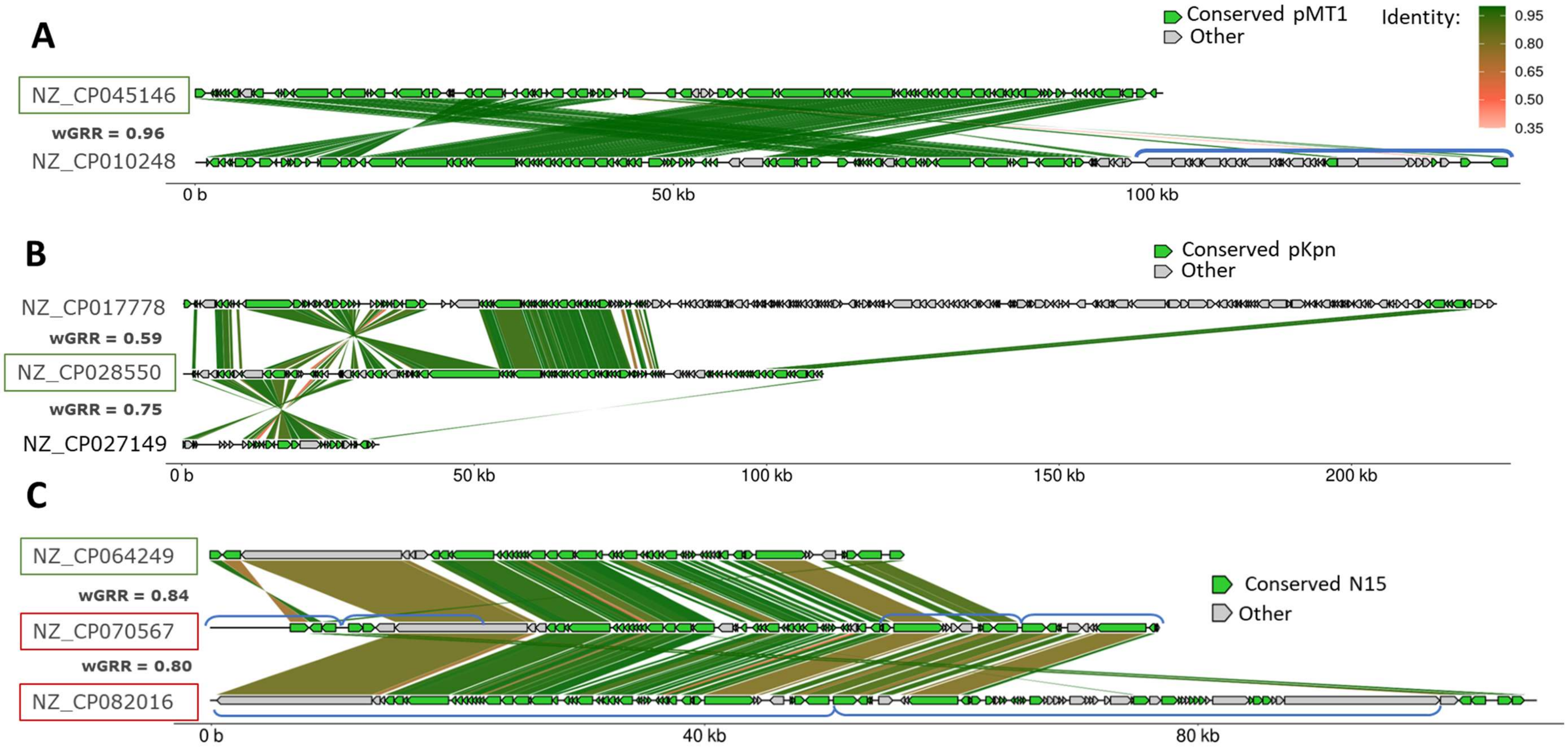

Figure S10. Examples of elements with atypical sizes. (A) pMT1-like P-P (high confident, green box) and a too-long element with additional sequences corresponding to conjugation module (indicated with blue brackets). (B) pKpn-like P-P (green box, NZ_CP028550), NZ_CP017778 (first row) is a too-long example and NZ_CP027149 (third row) is a too short example (detected only by MM-GRC). (C) Many of the N15-like cases (n=17, >72 kb) contain long inverted repeats of several kilobases. These repeats likely arose through wraparound reads that are commonly reported for linear elements if sequenced by PacBio (3).

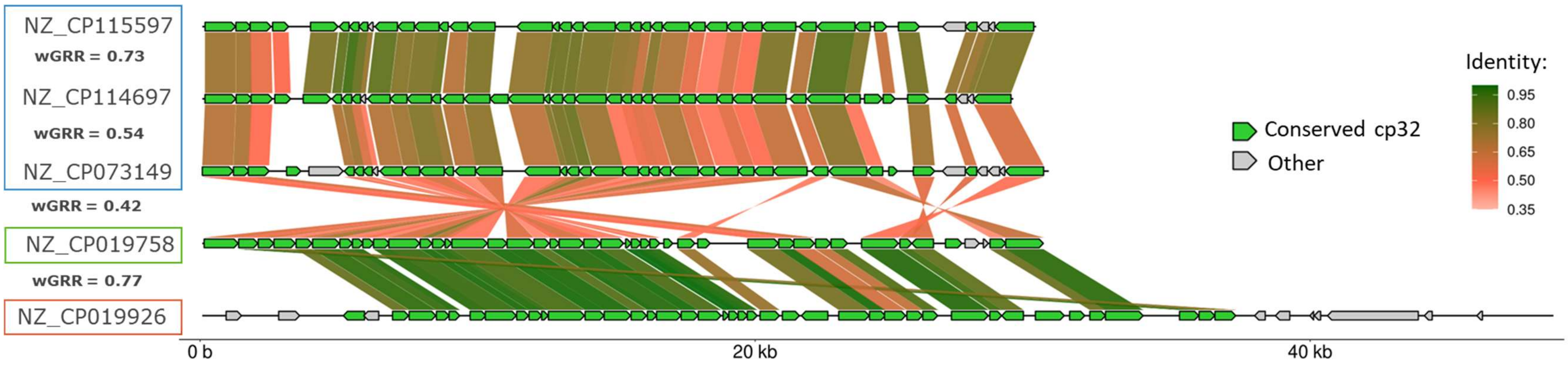

Figure S11. cp32 elements detected as P-Ps or excluded by tyPPing. cp32 P-P with high confidence (green box), an element with a too-long size (low confidence, red box), and three cp32-like P-Ps (medium confidence, blue box) from other hosts: NZ_CP115597 and NZ_CP114697 in *Borrelia miyamotoi*, and NZ_CP073149 in *Borrelia nietonii*. The cp32-like P-Ps (blue box) were only detected by tyPPing.

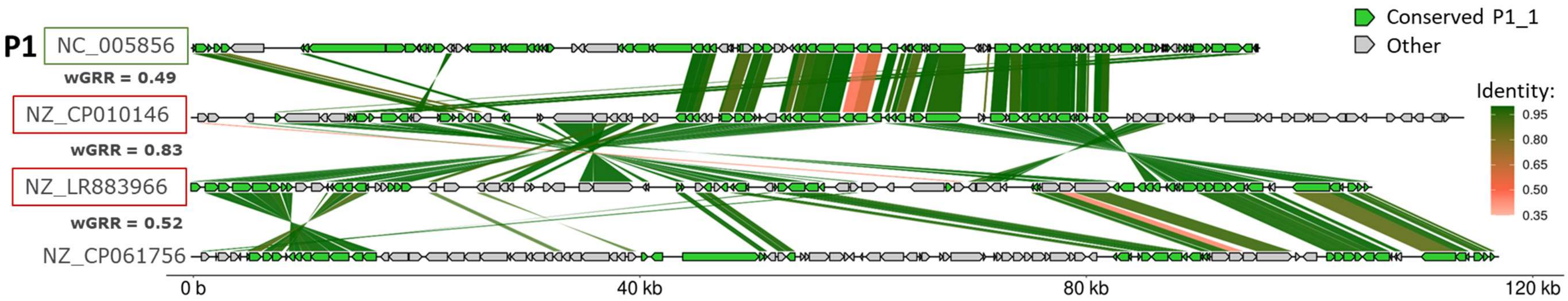

Figure S12. Detection of P1-like P-Ps of 03/21 by tyPPing. P1 (in green box) is compared to two elements (NZ_CP010146, NZ_LR883966) predicted by tyPPing to belong to P1_1 (medium confidence, red boxes). These elements were characterized as P1-like plasmids (4) that lack important phage genes and encode either *oriT* sequences or relaxases. NZ_CP061756, also a P1-like plasmid, was not detected as P-P by tyPPing.

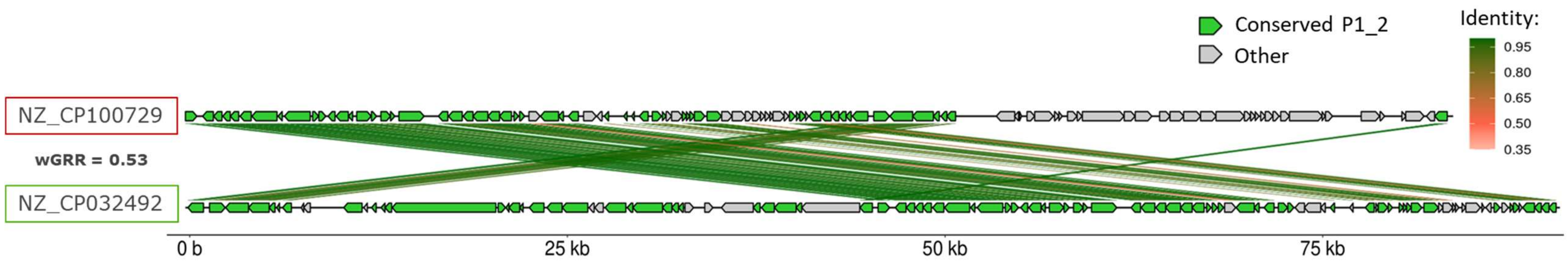

Figure S13. Questionable P1_2 element detected only by tyPPing in 05/23. P1_2 P-P (high confidence, green box) and an element predicted as P1_2 P-P (red box) by tyPPing. We speculate that this element is a plasmid, since it misses many P1_2 genes. Random forest models of MM-GRC assigned an average phage score of 0.496 (cutoff >0.5).

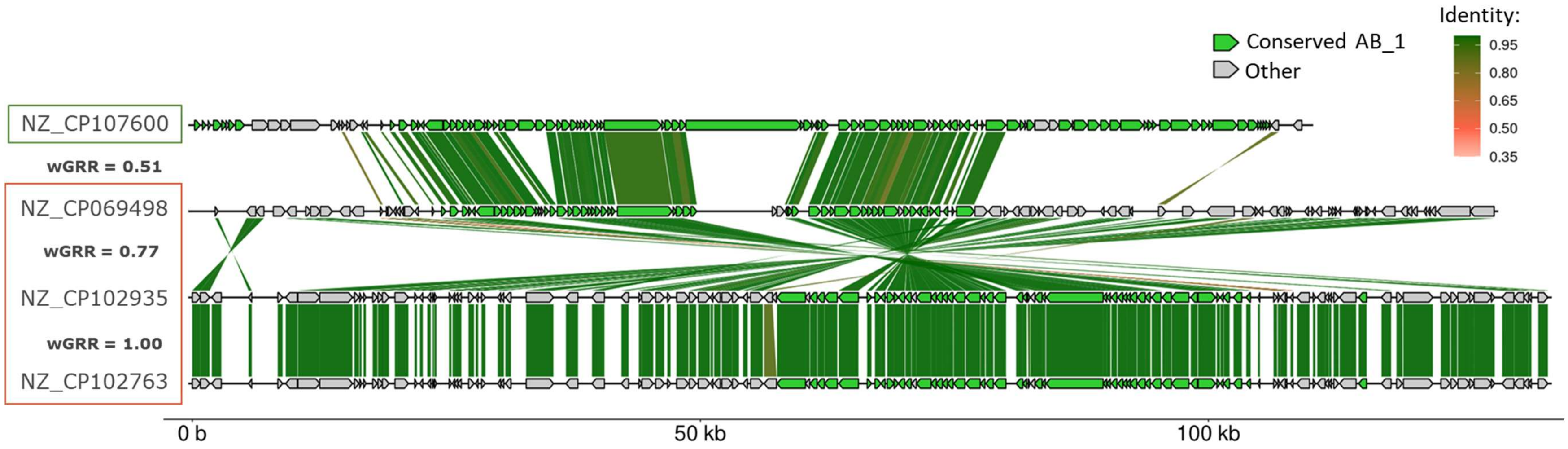

Figure S14: AB_1-like elements not detected by tyPPing. One high-confidence AB_1 (NZ_CP107600, green box) example and three cases (red boxes), detected by MM-GRC and not by tyPPing. These cases were not detected by tyPPing due to too few hits to AB_1 protein profiles.

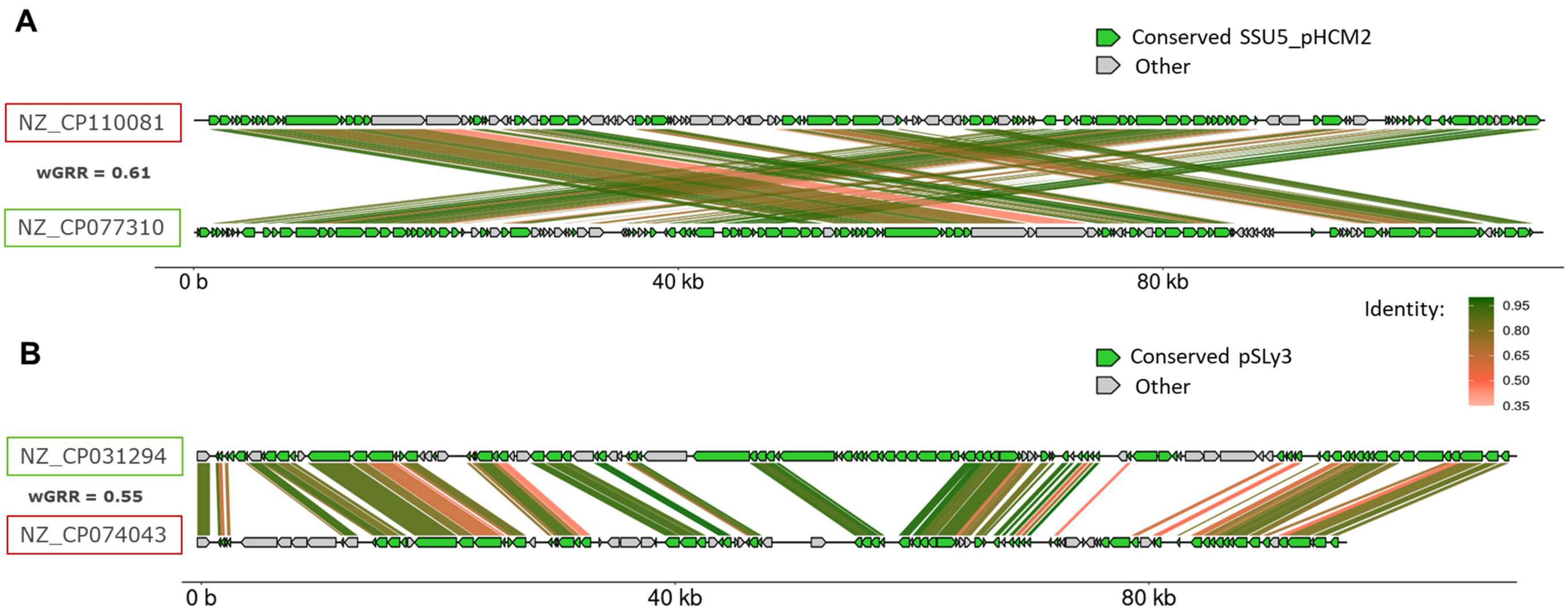

Figure S15: SSU5-like elements not detected by tyPPing. Two putative P-Ps, one SSU5_pHCM2-like (A, red box) and one pSLy3-like (B, red box), were missed by tyPPing A. SSU5_pHCM2-like P-P (high confidence, green box) and a similar P-P (NZ_CP110081, red box) detected in *Buttiauxella sp*. B. pSLy3-like P-P (high confidence, green box) and a related P-P (red box, NZ_CP074043) of *E. coli* strain PI6.

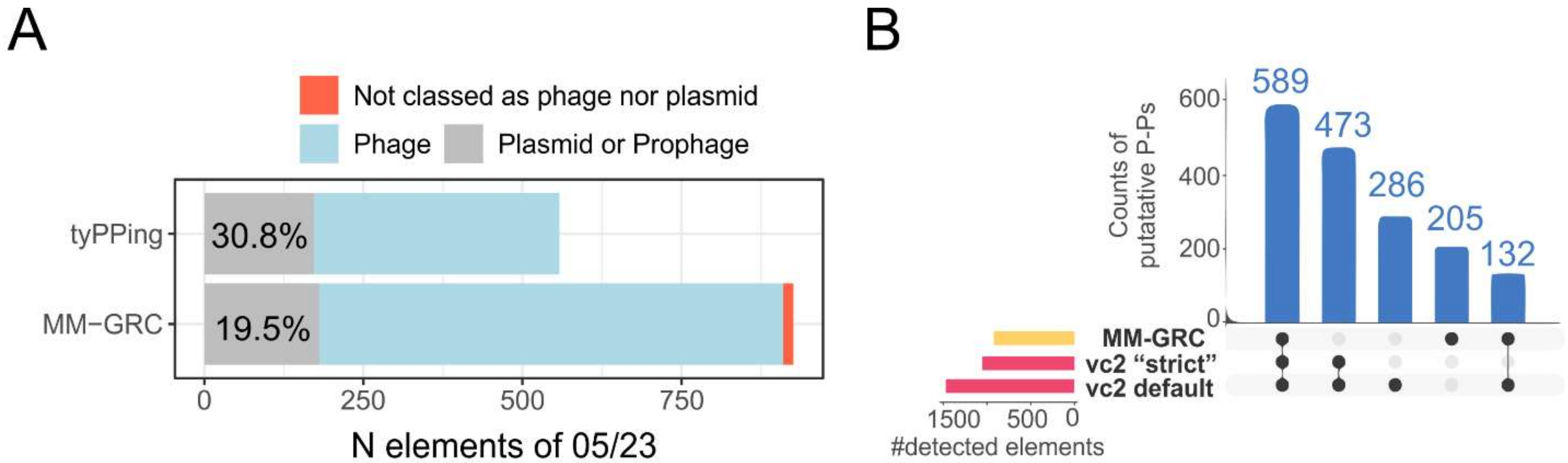

Figure S16. Classification of P-Ps of 05/23 using vConTACT v2 and geNomad. (A). Number of P-Ps predicted by tyPPing or MM-GRC classed by geNomad as phage (blue), plasmid and/or integrated prophage (grey) or not classed (red). **(B)** Counts of P-Ps predicted in 05/23 (not already present in 03/21), by using vConTACT v2 and 1416 P-Ps of 03/21 (4) as references. We compared two clustering approaches (‘strict’ and default) to predictions of MM-GRC. In ‘strict’ only cases that clearly clustered with P-Ps were considered as those. In default, cases that grouped in overlapping clusters or were categorized as Clustered/Singleton (described in the wiki of vConTACT v2 (5) https://bitbucket.org/MAVERICLab/vcontact2), were also considered.

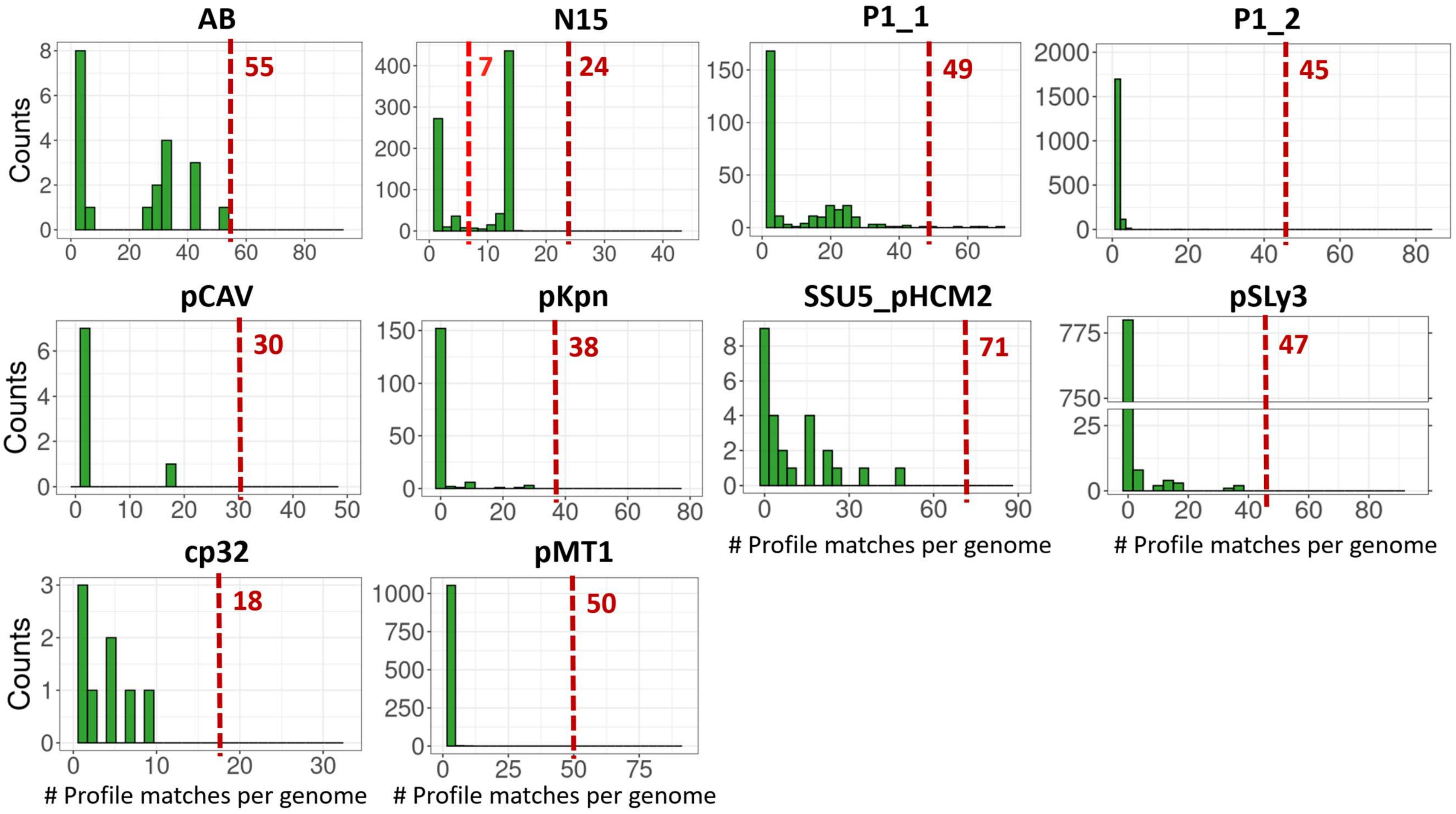

Figure S17. Number of matches (profile to protein) **per bacterial genome for each profile set.** tyPPings profiles were used to analyze 32 798 bacterial genomes that have plasmids and many most likely integrative prophages. Their counts were treated cumulative per genome (as in the tyPPing draft version), and if one profile matches several times, only the best hit is counted. P-Ps of any confidence were removed. Red dashed lines indicate MinProteins cutoffs. For N15, MinProteins uses 24 in the version for draft genomes, and 7 for complete sequences (see Methods).

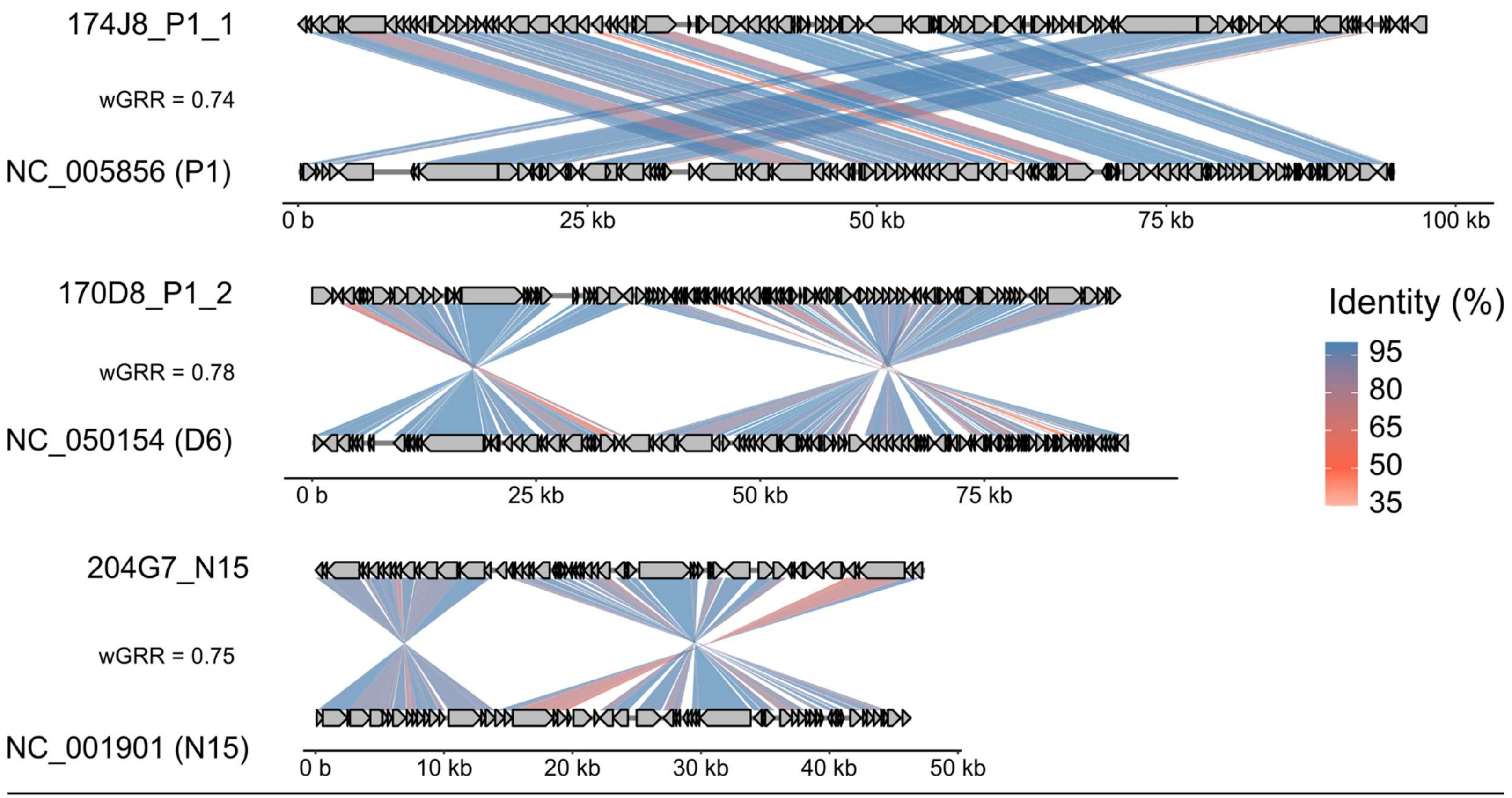

Figure S18. Complete assembled P-Ps of the CRE strains 174J8 (*E. coli*)**, 170D8** (*E. coli*) **and 204G7** (*E. cloacae*)**.** Circular, complete P-P genomes were assembled from long (PacBio) and short reads (following MMC induction, see Methods). These genomes were compared to P1 (NC005856), D6 (NC050154, P1_2), and N15 (NC001901) with gggenomes (https://github.com/thackl/gggenomes). Gene-to-gene assignments are BBHs. Pairwise protein similarity is given in %.

References

1. Faith,D.R., Kinnersley,M., Brooks,D.M., Drecktrah,D., Hall,L.S., Luo,E., Santiago-Frangos,A., Wachter,J., Samuels,D.S. and Secor,P.R. (2024) Characterization and genomic analysis of the Lyme disease spirochete bacteriophage ϕBB-1. *PLoS Pathog*, **20**, e1012122.

2. Steinegger,M., Meier,M., Mirdita,M., Vöhringer,H., Haunsberger,S.J. and Söding,J. (2019) HH-suite3 for fast remote homology detection and deep protein annotation. *BMC Bioinformatics*, **20**, 473.

3. Hepner,S., Kuleshov,K., Tooming-Kunderud,A., Alig,N., Gofton,A., Casjens,S., Rollins,R.E., Dangel,A., Mourkas,E., Sheppard,S.K., *et al.* (2023) A high fidelity approach to assembling the complex *Borrelia* genome. *BMC Genomics*, **24**, 401.

4. Pfeifer,E., Moura de Sousa,J.A., Touchon,M. and Rocha,E.P.C. (2021) Bacteria have numerous distinctive groups of phage-plasmids with conserved phage and variable plasmid gene repertoires. *Nucleic Acids Res*, **49**, 2655–2673.

5. Pfeifer,E. and Rocha,E.P.C. (2024) Phage-plasmids promote recombination and emergence of phages and plasmids. *Nat Commun*, **15**, 1545.

6. Bin Jang,H., Bolduc,B., Zablocki,O., Kuhn,J.H., Roux,S., Adriaenssens,E.M., Brister,J.R., Kropinski,A.M., Krupovic,M., Lavigne,R., *et al.* (2019) Taxonomic assignment of uncultivated prokaryotic virus genomes is enabled by gene-sharing networks. *Nat Biotechnol*, **37**, 632–639.
