# Supplementary figures and images for: Efficient detection and typing of phage-plasmids
Source: mBio. 2026 Feb 9;17(3):e03000-25. doi: 10.1128/mbio.03000-25 (PMC12977533; doi:10.1128/mbio.03000-25)

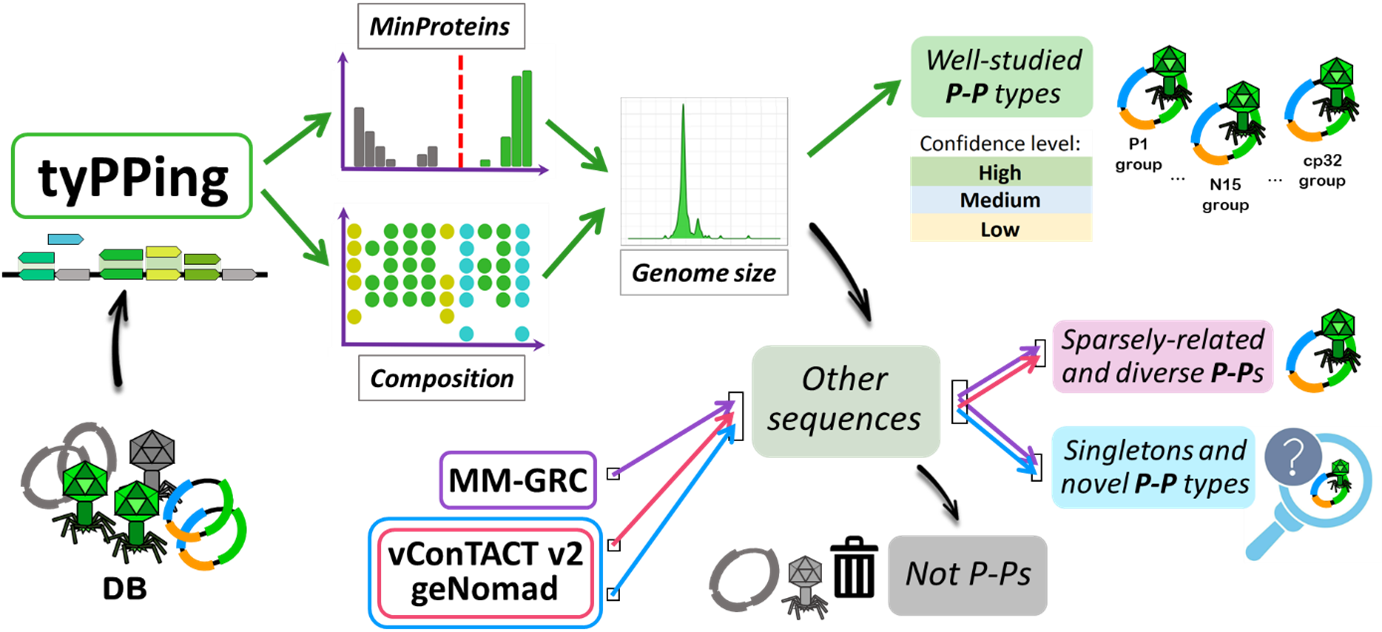

Supplement: Graphical abstract — Summary. [file mbio.03000-25-s0002.png]
